# Evaluating the effects of circulating inflammatory proteins as drivers and therapeutic targets for severe COVID-19

**DOI:** 10.3389/fimmu.2024.1352583

**Published:** 2024-02-22

**Authors:** Ancha Baranova, Jing Luo, Li Fu, Guanqun Yao, Fuquan Zhang

**Affiliations:** ^1^ School of Systems Biology, George Mason University, Manassas, VA, United States; ^2^ Research Centre for Medical Genetics, Moscow, Russia; ^3^ Department of Rheumatology, The Second Affiliated Hospital of Zhejiang University School of Medicine, Hangzhou, China; ^4^ School of Medicine, Tsinghua University, Beijing, China; ^5^ Department of Psychiatry, The Affiliated Brain Hospital of Nanjing Medical University, Nanjing, China; ^6^ School of Clinical Medicine, Tsinghua University, Beijing, China; ^7^ Institute of Neuropsychiatry, The Affiliated Brain Hospital of Nanjing Medical University, Nanjing, China

**Keywords:** circulating inflammatory protein, Mendelian randomization, COVID-19, GWAS, LIFR

## Abstract

**Objective:**

The relationships between circulating inflammatory proteins and COVID-19 have been observed in previous cohorts. However, it is not unclear which circulating inflammatory proteins may boost the risk of or protect against COVID-19.

**Methods:**

We performed Mendelian randomization (MR) analysis using GWAS summary result of 91 circulating inflammation-related proteins (N = 14,824) to assess their causal impact on severe COVID-19. The COVID-19 phenotypes encompassed both hospitalized (N = 2,095,324) and critical COVID-19 (N = 1,086,211). Moreover, sensitivity analyses were conducted to evaluate the robustness and reliability.

**Results:**

We found that seven circulating inflammatory proteins confer positive causal effects on severe COVID-19. Among them, serum levels of IL-10RB, FGF-19, and CCL-2 positively contributed to both hospitalized and critical COVID-19 conditions (OR: 1.10~1.16), while the other 4 proteins conferred risk on critical COVID-19 only (OR: 1.07~1.16), including EIF4EBP1, IL-7, NTF3, and LIF. Meanwhile, five proteins exert protective effects against hospitalization and progression to critical COVID-19 (OR: 0.85~0.95), including CXCL11, CDCP1, CCL4/MIP, IFNG, and LIFR. Sensitivity analyses did not support the presence of heterogeneity in the majority of MR analyses.

**Conclusions:**

Our study revealed risk and protective inflammatory proteins for severe COVID-19, which may have vital implications for the treatment of the disease.

## Introduction

Since the inception of SARS-CoV-2 spread through the globe, numerous risk factors for the severe course of COVID-19 have been identified, including obesity, cardiovascular diseases, diabetes, and certain respiratory conditions, including those induced by longtime smoking (1–4). In a majority of COVID-19 cases with pre-existing co-morbidities, the levels of cytokines and chemokines are perturbed in a profound fashion, to the degree that the term “cytokine storm” was coined.

Aberrant levels of circulating inflammatory proteins were repeatedly associated with more severe courses of COVID-19 in a variety of cohorts. In particular, at the early stage of illness, the signatures comprised of a combination of a low level of interferon type I (IFN-I) with elevated levels of proinflammatory cytokines, including tumor necrosis factor α (TNF-α), interleukin 6 (IL-6), IL-1β (IL-1β), C-X-C motif chemokine ligand 10 (CXCL10/IP10), macrophage inflammatory protein 1 alpha (MIP-1α), and chemokine (C-C motif) ligand 2 (CCL2) repeatedly displayed accuracy in predicting severe course of disease (5–7).

The Mendelian randomization (MR) framework is widely used to assess causality in disease etiology by utilizing genetic variants as instrumental variables (8). In this manner, one may assess possible causal relationships in a pair of environmental exposure (i.e. the level of a certain biomarker) and outcome (i.e. severe course of COVID-19). Because genetic variants are not a subject of the effect of any confounders and are randomized at meiosis, MR is analogous in its power of extracting causal relationships to randomized clinical trials (RCTs) (9). Thus, in recent years, MR has become an important analytical strategy to deal with sets of exposures and outcomes, when RCTs are impractical or unethical. Previous MR studies have confirmed the causal roles of pre-existing morbid conditions (2, 10, 11) and certain physiological and behavioral variables (12–14) in the severity of COVID-19, with many relevant insights extracted.

Other studies have revealed significant links between the state of the host’s immune response and COVID-19. Recent genome-wide association studies (GWAS) have identified 49 risk genes for severe COVID-19 cases (15, 16). Additionally, the effects of the IFNAR2 and IL10RB gene products in the COVID-19 affected lungs were detected (17). Many of the highlighted genes encode various components of immune response circuits and pro-inflammatory cascades.

Some attempts were also made to discern the causal relationship between soluble proteins and COVID-19; the scope of these studies, however, remained relatively limited. For example, Sun Y et al. detected a set of circulating proteins associated with an increased risk of or protection from severe COVID-19, yet a detailed analysis of their specific roles was not performed (18). Another study has described the relationship between immune system-mediated aging represented by lower counts of CD19+ B-cells, the activity of the Notch pathway, and the risk of contracting COVID-19 (19), but the proteins profiled in the frame of the study were limited to that represented by in clinical tests or biological age signatures. Therefore, the risks or protections provided by circulating proteins against severe forms of COVID remain understudied.

So far, only one study systematically analyzed causal associations between steady-state levels of circulating molecules and COVID-19 outcomes (20). While a number of meaningful casual associations were recovered, this previous study has profiled twice a smaller set of molecules in a smaller population. Therefore, we sought to test the potential causal effects of 91 circulating inflammatory proteins on severe COVID-19 in the largest eQTL and COVID-19 outcomes datasets available to date.

## Materials and methods

Publicly available GWAS summary results were used in this study. The GWAS summary data for 91 plasma inflammatory proteins were derived from the measurements made on the Olink platform in 14,824 participants (21). We obtained two GWAS datasets on severe COVID-19 from the COVID-19 Host Genetic Initiative (HGI) GWAS (round 7, the European subset) (22), including the hospitalized COVID-19 (32,519 hospitalized patients and 2,062,805 controls) and the critical COVID-19 (13,769 critically ill patients and 1,072,442 controls).

Causal effects were assessed by the inverse variance weighting (IVW) model along with the weighted median (WM) and MR-Egger models as complementary measures to ensure sensitivity (23). The WM and MR-Egger models are typically less powerful than the IVW model but perform better in the case of horizontal pleiotropy or invalid instruments. Potential horizontal pleiotropy was determined by the intercept of the MR-Egger regression and heterogeneity by both Cochran’s Q test (P < 0.05) and I^2^ statistics (I^2^ > 0.25). These MR methods were implemented in the TwoSampleMR package (version 0.5.6) (23).

Within each MR analysis, single nucleotide polymorphisms (SNPs) associated with each circulating protein (P < 1 × 10^-5^) were selected. These SNPs were further pruned by a clumping r^2^ value of 0.001 within a 10 Mb window and were used as instrumental variables (IVs).

## Results

**Figure 1:**
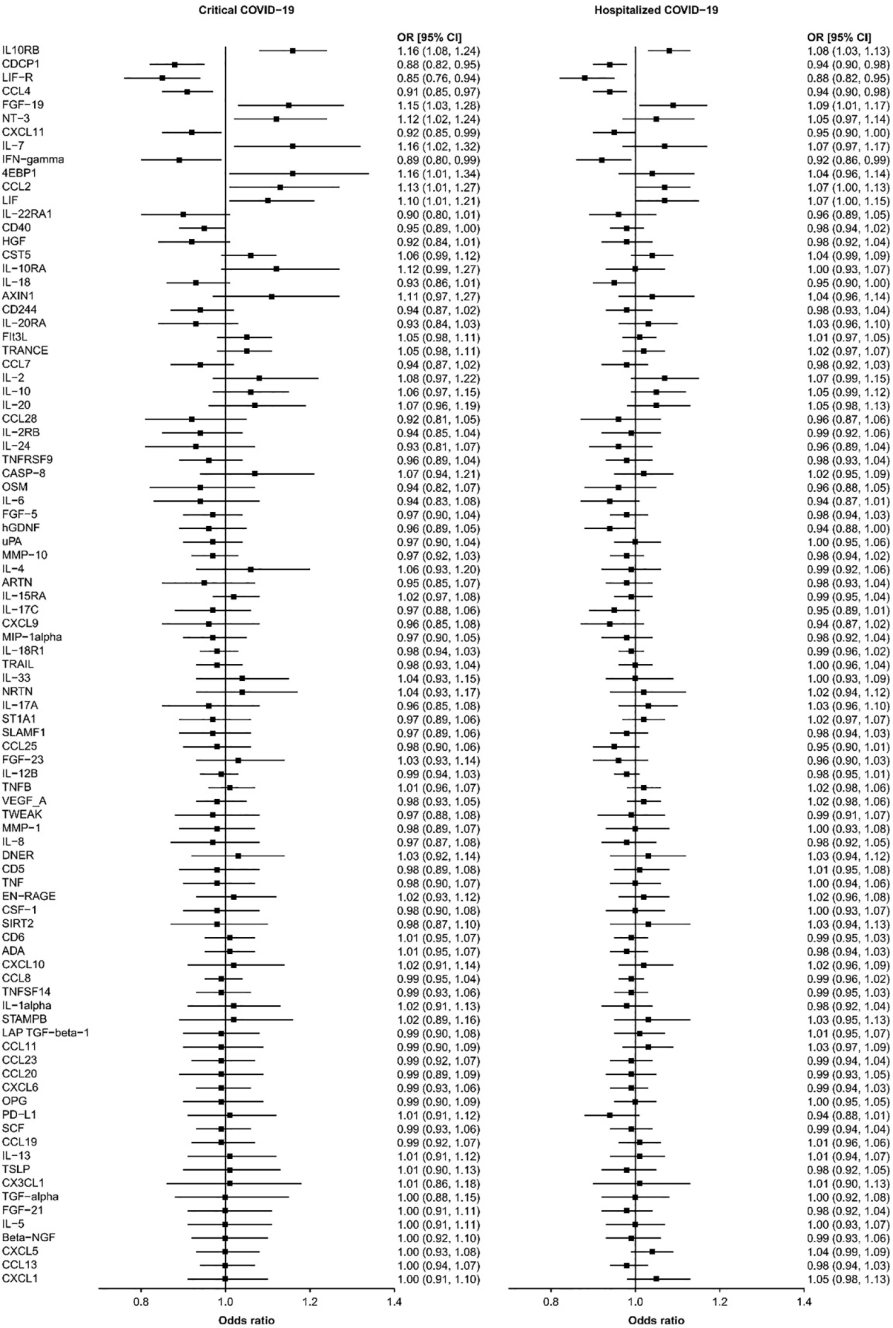
The causal relationships between the 91 circulating proteins and COVID-19. The forest plot showed the odds ratio (OR) and 95% confidence interval (CI) results of inverse-variance weighted MR for the relationships between the 91 circulating proteins and critical and hospitalized COVID-19.

We found that seven circulating inflammatory proteins confer positive causal effects on severe COVID-19, while five circulating inflammatory proteins protected against severe forms of this disorder. Among the seven risk-increasing proteins, Interleukin-10 (IL-10) Receptor subunit β, FGF-19, and CCL-2 were positively and causally contributing to both hospitalized COVID-19 and critical COVID-19 (OR: 1.10~1.16), while eukaryotic translation initiation factor 4E binding protein 1 (EIF4EBP1), IL-7, Neurotrophin-3 (NTF3), and Leukemia Inhibitory Factor (LIF) served as causal risk factors for critical COVID-19 only (OR: 1.07~1.16). All the five protective proteins, namely, C-X-C motif chemokine 11 (CXCL11), CUB domain-containing protein 1 (CDCP1), macrophage inflammatory protein CCL4/MIP, Interferon Gamma (IFNG) and leukemia inhibitory factor receptor (LIFR), were associated with a decrease in the risks of being hospitalized with COVID-19 and of developing critical COVID-19 (OR: 0.85~0.95) (**Table 1** and **Figure 1**).

**Table 1:** Causal effects of circulating inflammatory proteins on COVID-19.

Exposure	Outcome	N_IV	b (se)	OR [95%CI]	P	FDR
EIF4EBP1	Critical COVID-19	17	0.153 (0.072)	1.16 [1.01-1.34]	0.033	0.362
IL7	Critical COVID-19	23	0.150 (0.066)	1.16 [1.02-1.32]	0.023	0.354
IL10RB	Critical COVID-19	29	0.147 (0.034)	1.16 [1.08-1.24]	1.67E-05	0.005
IL10RB	Hospitalized COVID-19	29	0.075 (0.023)	1.08 [1.03-1.13]	1.23E-03	0.067
FGF19	Critical COVID-19	33	0.141 (0.054)	1.15 [1.03-1.28]	9.64E-03	0.188
FGF19	Hospitalized COVID-19	33	0.082 (0.036)	1.09 [1.01-1.17]	0.025	0.360
CCL2	Critical COVID-19	30	0.123 (0.058)	1.13 [1.01-1.27]	0.033	0.362
CCL2	Hospitalized COVID-19	30	0.064 (0.030)	1.07 [1.00-1.13]	0.034	0.362
NTF3	Critical COVID-19	31	0.117 (0.049)	1.12 [1.02-1.24]	0.016	0.271
LIF	Critical COVID-19	27	0.098 (0.047)	1.10 [1.01-1.21]	0.038	0.364
CXCL11	Critical COVID-19	38	-0.089 (0.039)	0.92 [0.85-0.99]	0.022	0.354
CXCL11	Hospitalized COVID-19	38	-0.052 (0.025)	0.95 [0.90-1.00]	0.037	0.364
CDCP1	Critical COVID-19	33	-0.125 (0.036)	0.88 [0.82-0.95]	4.27E-04	0.039
CDCP1	Hospitalized COVID-19	34	-0.061 (0.024)	0.94 [0.90-0.98]	9.15E-03	0.188
CCL4	Critical COVID-19	30	-0.098 (0.034)	0.91 [0.85-0.97]	3.69E-03	0.101
CCL4	Hospitalized COVID-19	30	-0.062 (0.020)	0.94 [0.90-0.98]	1.66E-03	0.075
IFNG	Critical COVID-19	20	-0.121 (0.054)	0.89 [0.80-0.99]	0.026	0.360
IFNG	Hospitalized COVID-19	19	-0.081 (0.038)	0.92 [0.86-0.99]	0.032	0.362
LIFR	Critical COVID-19	29	-0.164 (0.053)	0.85 [0.76-0.94]	1.99E-03	0.078
LIFR	Hospitalized COVID-19	29	-0.124 (0.037)	0.88 [0.82-0.95]	7.50E-04	0.051

OR, odds ratio; CI, confidence interval; b, effect size; se, standard error; N_IV, number of instrumental variables.

The sensitivity analyses indicated that the directions of causal effects across the methods were largely the same (**Supplementary Table 1**). The MR-Egger regression test did not support the directional pleiotropy in the majority of MR analyses (MR-Egger intercept < 0.01, P > 0.05). The Cochran Q test and I^2^ statistic did not support the existence of heterogeneity in most MR analyses.

## Discussion

In this study, we conducted an MR analysis to explore the causality between the levels of various circulating proteins and COVID-19. The list of circulation proteins profiled in this study included a total of 91 molecules and, therefore, was substantially larger than the typical set of 10 to 30 cytokines and chemokines covered by soluble protein assays in clinical studies of SARS-CoV-2 infections. Our results indicated that some proteins were indeed associated with an increased risk of severe COVID.

Some of these proteins were never associated with a pathophysiology or a severe course of SARS-CoV-2 before. In particular, we found that the level of LIF and its soluble receptor LIFR were negatively co-regulated; the genetic components defining higher levels of LIF and lower levels of LIFR served as causal contributors to the severity of COVID-19 phenotypes.

LIF belongs to the IL-6 cytokine family. Through its binding to a receptor complex consisting of gp130 and LIFR, both of which are constitutively associated with receptor-associated JAK molecules, particularly JAK1, LIF exerts pleiotropic effects on many cellular types (24). LIFR is abundant in the plasmacytoid dendritic cells (pDCs), which produce abundant type I IFNs (IFN-I) in response to viral nucleic acids. When stimulated with LIF, pDCs suppress their responses to CpG through inhibition of IFN-I and NF-κB signaling. In a nutshell, the LIF signal renders both pDCs and late DC progenitors refractory to physiological stimuli controlling pDC functions and development (25), and, therefore, delays proper immunological response to viral infections, including SARS-CoV-2. It is important to note that the genome-wide protein quantitative trait locus (pQTL) Olink dataset covered only soluble molecules and, therefore, reported the levels of soluble isoform of LIFR (sLIFR) (26), which serves as a molecular decoy, or antagonist, which binds to and neutralizes LIF in plasma by titration. Therefore, the severe course of SARS-CoV-2 would be expected when levels of LIF are higher, and levels of soluble isoform of LIFR would be lower, in accordance with our observations.

The majority of other molecules genetically connected to the severity of COVID-19 have been either described as possible participants in its pathophysiology or their potential links have been suggested in previous observations. The directionality of the effects, as they are observed in our study, should be interpreted with certain cautions, as the design of the eQTL study grasps the steady-state levels of certain soluble molecules, while observational studies profiling same molecules in patients with severe forms of COVID-19 show the relative induction of these molecules, with a larger magnitude of the change achieved in patients who started at lower levels. For example, soluble proteins IFN-γ, CXCL11, and CCL4, all three being associated with lower chances of developing severe forms of SARS-CoV-2 when elevated, are well-known as being connected to each other in a coregulated fashion, and, therefore, form a functional unit. In this unit, IFN-γ, a pleiotropic cytokine with roles in a variety of biological responses including protection from viral and bacterial infections, induces both CXCL11 and CCL4 (27), and, by doing that, promotes cytokine storm. In some antiviral therapeutic approaches, co-inducible inflammatory cytokines are even treated as a collective pharmacological target (28). When, at the inception of the disease, levels of both the IFN-γ and the secondary cytokines/chemokines are too low, such as pre-existing suppression, the inflammatory cascade response intended to counteract viral replication may develop further after a certain delay, and eventually progress to a detrimental extent (29).

Alternatively, observed rises in the levels of certain protective molecules may be compensatory in their nature. In this way, causal associations of a higher level of CDCP1 with a decrease in the risks of being hospitalized with COVID-19 and of developing critical COVID-19 does not contradict an observation that elevation of this biomarker was also found in the serum of patients recovering from the most severe forms of COVID-19 at a time point of 45 days after a discharge from a hospital (30). As CDCP1 negatively regulates TGF-β signaling and myofibroblast differentiation (31), one may hypothesize that a larger magnitude of TGF-beta deregulation may be counterbalanced by stronger engagement of CDCP1 when homeostatic control loops are attempting to compensate.

Among the molecules that causally and positively contributed to the severity of COVID-19, some were previously highlighted in genome-wide as potential contributors either to COVID-19-related hospitalizations or to SARS-CoV-2 infection susceptibility. In particular, this relates to proteins encoded by *IL10RB* (2, 32, 33), *CCL2* (34, 35), and *IL7* (36, 37) loci. For example, carriers of the rs9976829 variant which is located close to the *IL-10RB* locus have a higher susceptibility to COVID-19. Indeed, IL-10RB plays a key in regulating the macrophage and monocyte; moreover, its targeting by several interferon-related drugs aids in recovery from COVID-19 (17). Much is also known about CCL2, also known as monocyte chemotactic protein-1 (MCP-1). Through its receptor CCR2, this chemokine orchestrates an excessive inflammatory response by recruiting leukocytes into the lung tissue and, therefore, driving the progression of COVID-19 to critical lung injury (35). In the case of IL7, its elevation was shown to correlate with COVID-19 severity directly, with exhaustion of T cells being a possible mediator (38).

Less is known about connections between phenotypes of COVID-19 and the levels of FGF-19, EIF4EBP1, and Neurotrophin-3 (NTF3). FGF-19 plays an important role in the maintenance of the enterohepatic bile acid/cholesterol system (39). Through the reduction of liver fat content and plasma glucose and the improvement of the lipid formula of the blood, FGF19 protects against metabolic syndrome (40). In our study, higher steady-state levels of FGF19 were positively contributing to both analyzed outcomes, hospitalized COVID-19 and critical COVID-19. This seemingly contradictory finding may be explained by the mechanism, by which FGF-19 achieves compensiation for metabolic shifts. Treatments with FGF-19 substantially increase fatty acid oxidation and Body Metabolic Rate (BMR) (41). In turn, higher BMRs positively and causally contribute to severe COVID-19, according to another of our analyses using MR (12).

EIF4EBP1 is a substrate of mTOR and activated EKR, in response to its phosphorylation, EIF4EBP1 aids ribosomes in ramping up protein production (42). In cell-based experiments, levels of EIF4EBP1 rise along with an increase in the production of SARS-CoV-2 virions (43). Respectively, mTOR inhibitors, including metformin, which shut down activation of the translation factor 4E-BP may suppress viral replication (44). On the other hand, many human-infecting viruses selectively shut down the translation of the host proteins by destroying 4E-BP factors, thus, skewing the outputs of protein-producing machinery to their end. Due to the lack of a role of 4E-BP studies in SARS-CoV-2-related models, the causal link between the increase in the plasma levels of EIF4EBP1 and a severe SARS-CoV-infection is hard to interpret from the functional point of view. However, one may surmise that these intracellular functions of EIF4EBP1 may not be relevant to its levels in plasma, which were the ones measured in the Olink-based eQTL datasets employed in the current work. Instead, these levels may reflect the rates of cellular death and the subsequent release of this cytoplasmic factor, or some new, not yet described function of EIF4EBP1, not related to intracellular protein biosynthesis.

Along with other neurotrophins, NTF3 plays roles in the central nervous system (CNS), but also in immune cell regulating, promoting the survival of monocytes and lymphocytes, and influencing cytokine expression. In CNS, NT-3 binds to and activates TrkC, but also, to a lesser degree, other Trk receptors (45). Most plausibly, observations of the causal association of circulating NT-3 levels with the severity of SARS-CoV-2 could be explained by peripheral TrkC signaling, for example, one involved in migration maturation of the mast cell precursors (46).

Our study has some limitations. On one hand, to reduce population heterogeneity, we restricted our study to individuals of European descent; therefore, care should be taken to extrapolate our results into other populations. On the other hand, after conducting FDR correction, most MR results no longer exhibited statistical significance. It is necessary to validate some findings in additional datasets in the future.

## Conclusions

In summary, our study supports that genetically-defined steady-state levels of certain circulating proteins may increase the risk of or protect against severe forms of COVID-19. The dissection of the respective molecular pathways and their pharmacological targeting may improve the outcomes of COVID-19 in individuals infected with SARS-CoV-2.

## Data availability statement

The original contributions presented in the study are included in the article/**Supplementary Material**. Further inquiries can be directed to the corresponding author.

## Author contributions

AB: Writing – original draft, Writing – review & editing. JL: Writing – original draft, Writing – review & editing. LF: Writing – original draft, Writing – review & editing. GY: Writing – review & editing. FZ: Writing – original draft, Writing – review & editing, Conceptualization, Formal Analysis, Software, Supervision.
