# Supplementary material for: Evaluating the effects of circulating inflammatory proteins as drivers and therapeutic targets for severe COVID-19
Source: Front Immunol. 2024 Feb 22;15:1352583. doi: 10.3389/fimmu.2024.1352583 (PMC10917991; doi:10.3389/fimmu.2024.1352583)
Supplement: Supplementary file 1 [file Table_1.docx]

**Supplementary Table 1****. The causal effects of circulating inflammatory proteins on COVID-19 by three methods.**

| **Exposure** | **Protein** | **Outcome** | **Method** | **N_IV** | **b (se)** | **OR [95%CI]** | **Q** | **Q_P** | **I2** | **Egger_intercept** | **P_pleiotropy** | **P** |
| --- | --- | --- | --- | --- | --- | --- | --- | --- | --- | --- | --- | --- |
| GCST90274758 | 4EBP1 | Hospitalized COVID-19 | IVW | 18 | 0.043 (0.043) | 1.04 [0.96-1.14] | 23.64 | 0.129 | 0.281 | NA | NA | 0.31 |
| GCST90274758 | 4EBP1 | Hospitalized COVID-19 | WM | 18 | -0.030 (0.054) | 0.97 [0.87-1.08] | NA | NA | NA | NA | NA | 0.57 |
| GCST90274758 | 4EBP1 | Hospitalized COVID-19 | MR-Egger | 18 | 0.066 (0.109) | 1.07 [0.86-1.32] | 23.57 | 0.099 | 0.279 | -0.002 | 0.824 | 0.554 |
| GCST90274759 | ADA | Hospitalized COVID-19 | IVW | 25 | -0.017 (0.023) | 0.98 [0.94-1.03] | 37.96 | 0.035 | 0.368 | NA | NA | 0.457 |
| GCST90274759 | ADA | Hospitalized COVID-19 | WM | 25 | -0.018 (0.023) | 0.98 [0.94-1.03] | NA | NA | NA | NA | NA | 0.437 |
| GCST90274759 | ADA | Hospitalized COVID-19 | MR-Egger | 25 | -0.015 (0.029) | 0.98 [0.93-1.04] | 37.94 | 0.026 | 0.367 | -0.001 | 0.912 | 0.603 |
| GCST90274760 | ARTN | Hospitalized COVID-19 | IVW | 32 | -0.017 (0.030) | 0.98 [0.93-1.04] | 31.92 | 0.421 | 0.029 | NA | NA | 0.582 |
| GCST90274760 | ARTN | Hospitalized COVID-19 | WM | 32 | -0.024 (0.042) | 0.98 [0.90-1.06] | NA | NA | NA | NA | NA | 0.569 |
| GCST90274760 | ARTN | Hospitalized COVID-19 | MR-Egger | 32 | -0.048 (0.068) | 0.95 [0.83-1.09] | 31.64 | 0.384 | 0.02 | 0.003 | 0.611 | 0.488 |
| GCST90274761 | AXIN1 | Hospitalized COVID-19 | IVW | 13 | 0.044 (0.045) | 1.04 [0.96-1.14] | 10.12 | 0.605 | -0.186 | NA | NA | 0.329 |
| GCST90274761 | AXIN1 | Hospitalized COVID-19 | WM | 13 | 0.048 (0.061) | 1.05 [0.93-1.18] | NA | NA | NA | NA | NA | 0.428 |
| GCST90274761 | AXIN1 | Hospitalized COVID-19 | MR-Egger | 13 | 0.084 (0.114) | 1.09 [0.87-1.36] | 9.98 | 0.533 | -0.203 | -0.004 | 0.711 | 0.479 |
| GCST90274762 | β-NGF | Hospitalized COVID-19 | IVW | 32 | -0.007 (0.033) | 0.99 [0.93-1.06] | 32.53 | 0.391 | 0.047 | NA | NA | 0.841 |
| GCST90274762 | β-NGF | Hospitalized COVID-19 | WM | 32 | 0.009 (0.046) | 1.01 [0.92-1.11] | NA | NA | NA | NA | NA | 0.839 |
| GCST90274762 | β-NGF | Hospitalized COVID-19 | MR-Egger | 32 | -0.003 (0.073) | 1.00 [0.86-1.15] | 32.53 | 0.343 | 0.047 | 0 | 0.956 | 0.969 |
| GCST90274763 | CASP-8 | Hospitalized COVID-19 | IVW | 22 | 0.019 (0.034) | 1.02 [0.95-1.09] | 20.14 | 0.512 | -0.043 | NA | NA | 0.586 |
| GCST90274763 | CASP-8 | Hospitalized COVID-19 | WM | 22 | -0.014 (0.051) | 0.99 [0.89-1.09] | NA | NA | NA | NA | NA | 0.789 |
| GCST90274763 | CASP-8 | Hospitalized COVID-19 | MR-Egger | 22 | 0.032 (0.079) | 1.03 [0.88-1.21] | 20.11 | 0.451 | -0.044 | -0.001 | 0.857 | 0.693 |
| GCST90274764 | CCL11 | Hospitalized COVID-19 | IVW | 29 | 0.030 (0.030) | 1.03 [0.97-1.09] | 35.59 | 0.153 | 0.213 | NA | NA | 0.327 |
| GCST90274764 | CCL11 | Hospitalized COVID-19 | WM | 29 | 0.024 (0.043) | 1.02 [0.94-1.11] | NA | NA | NA | NA | NA | 0.569 |
| GCST90274764 | CCL11 | Hospitalized COVID-19 | MR-Egger | 29 | 0.048 (0.062) | 1.05 [0.93-1.18] | 35.44 | 0.128 | 0.21 | -0.002 | 0.736 | 0.444 |
| GCST90274765 | CCL19 | Hospitalized COVID-19 | IVW | 37 | 0.006 (0.026) | 1.01 [0.96-1.06] | 39.76 | 0.306 | 0.095 | NA | NA | 0.806 |
| GCST90274765 | CCL19 | Hospitalized COVID-19 | WM | 37 | -0.014 (0.038) | 0.99 [0.92-1.06] | NA | NA | NA | NA | NA | 0.702 |
| GCST90274765 | CCL19 | Hospitalized COVID-19 | MR-Egger | 37 | -0.013 (0.049) | 0.99 [0.90-1.09] | 39.51 | 0.275 | 0.089 | 0.003 | 0.638 | 0.793 |
| GCST90274766 | CCL20 | Hospitalized COVID-19 | IVW | 33 | -0.015 (0.031) | 0.99 [0.93-1.05] | 33.89 | 0.377 | 0.056 | NA | NA | 0.627 |
| GCST90274766 | CCL20 | Hospitalized COVID-19 | WM | 33 | -0.006 (0.045) | 0.99 [0.91-1.08] | NA | NA | NA | NA | NA | 0.887 |
| GCST90274766 | CCL20 | Hospitalized COVID-19 | MR-Egger | 33 | -0.186 (0.077) | 0.83 [0.71-0.97] | 28.03 | 0.62 | -0.142 | 0.016 | 0.022 | 0.021 |
| GCST90274767 | CCL23 | Hospitalized COVID-19 | IVW | 35 | -0.012 (0.026) | 0.99 [0.94-1.04] | 53.01 | 0.02 | 0.359 | NA | NA | 0.629 |
| GCST90274767 | CCL23 | Hospitalized COVID-19 | WM | 35 | -0.036 (0.027) | 0.96 [0.92-1.02] | NA | NA | NA | NA | NA | 0.17 |
| GCST90274767 | CCL23 | Hospitalized COVID-19 | MR-Egger | 35 | -0.045 (0.037) | 0.96 [0.89-1.03] | 50.67 | 0.025 | 0.329 | 0.007 | 0.225 | 0.227 |
| GCST90274768 | CCL25 | Hospitalized COVID-19 | IVW | 33 | -0.047 (0.029) | 0.95 [0.90-1.01] | 84.44 | 1.30E-06 | 0.621 | NA | NA | 0.11 |
| GCST90274768 | CCL25 | Hospitalized COVID-19 | WM | 33 | 0.004 (0.026) | 1.00 [0.95-1.06] | NA | NA | NA | NA | NA | 0.873 |
| GCST90274768 | CCL25 | Hospitalized COVID-19 | MR-Egger | 33 | -0.076 (0.045) | 0.93 [0.85-1.01] | 82.55 | 1.43E-06 | 0.612 | 0.005 | 0.406 | 0.105 |
| GCST90274769 | CCL28 | Hospitalized COVID-19 | IVW | 38 | -0.044 (0.051) | 0.96 [0.87-1.06] | 1.11E+02 | 2.64E-09 | 0.666 | NA | NA | 0.386 |
| GCST90274769 | CCL28 | Hospitalized COVID-19 | WM | 38 | -0.019 (0.043) | 0.98 [0.90-1.07] | NA | NA | NA | NA | NA | 0.656 |
| GCST90274769 | CCL28 | Hospitalized COVID-19 | MR-Egger | 38 | -0.006 (0.107) | 0.99 [0.81-1.23] | 1.10E+02 | 1.76E-09 | 0.665 | -0.004 | 0.688 | 0.955 |
| GCST90274770 | CCL4 | Hospitalized COVID-19 | IVW | 30 | -0.062 (0.020) | 0.94 [0.90-0.98] | 31.66 | 0.335 | 0.084 | NA | NA | 1.66E-03 |
| GCST90274770 | CCL4 | Hospitalized COVID-19 | WM | 30 | -0.107 (0.027) | 0.90 [0.85-0.95] | NA | NA | NA | NA | NA | 9.61E-05 |
| GCST90274770 | CCL4 | Hospitalized COVID-19 | MR-Egger | 30 | -0.090 (0.027) | 0.91 [0.87-0.96] | 29.48 | 0.388 | 0.016 | 0.007 | 0.161 | 2.78E-03 |
| GCST90274771 | CD244 | Hospitalized COVID-19 | IVW | 32 | -0.019 (0.029) | 0.98 [0.93-1.04] | 40.37 | 0.121 | 0.232 | NA | NA | 0.512 |
| GCST90274771 | CD244 | Hospitalized COVID-19 | WM | 32 | 0.047 (0.041) | 1.05 [0.97-1.14] | NA | NA | NA | NA | NA | 0.245 |
| GCST90274771 | CD244 | Hospitalized COVID-19 | MR-Egger | 32 | 0.084 (0.055) | 1.09 [0.98-1.21] | 35.05 | 0.241 | 0.115 | -0.013 | 0.041 | 0.139 |
| GCST90274772 | CD40 | Hospitalized COVID-19 | IVW | 25 | -0.023 (0.021) | 0.98 [0.94-1.02] | 14.06 | 0.945 | -0.706 | NA | NA | 0.272 |
| GCST90274772 | CD40 | Hospitalized COVID-19 | WM | 25 | -0.009 (0.026) | 0.99 [0.94-1.04] | NA | NA | NA | NA | NA | 0.724 |
| GCST90274772 | CD40 | Hospitalized COVID-19 | MR-Egger | 25 | -0.013 (0.030) | 0.99 [0.93-1.05] | 13.82 | 0.932 | -0.736 | -0.002 | 0.627 | 0.676 |
| GCST90274773 | CD5 | Hospitalized COVID-19 | IVW | 32 | 0.013 (0.032) | 1.01 [0.95-1.08] | 40.84 | 0.111 | 0.241 | NA | NA | 0.684 |
| GCST90274773 | CD5 | Hospitalized COVID-19 | WM | 32 | -0.032 (0.042) | 0.97 [0.89-1.05] | NA | NA | NA | NA | NA | 0.439 |
| GCST90274773 | CD5 | Hospitalized COVID-19 | MR-Egger | 32 | -0.020 (0.079) | 0.98 [0.84-1.14] | 40.56 | 0.095 | 0.236 | 0.004 | 0.65 | 0.8 |
| GCST90274774 | CD6 | Hospitalized COVID-19 | IVW | 27 | -0.011 (0.018) | 0.99 [0.95-1.03] | 26.48 | 0.437 | 0.018 | NA | NA | 0.557 |
| GCST90274774 | CD6 | Hospitalized COVID-19 | WM | 27 | -0.017 (0.023) | 0.98 [0.94-1.03] | NA | NA | NA | NA | NA | 0.477 |
| GCST90274774 | CD6 | Hospitalized COVID-19 | MR-Egger | 27 | -0.004 (0.027) | 1.00 [0.95-1.05] | 26.34 | 0.39 | 0.013 | -0.002 | 0.723 | 0.884 |
| GCST90274775 | CDCP1 | Hospitalized COVID-19 | IVW | 34 | -0.061 (0.024) | 0.94 [0.90-0.98] | 31.27 | 0.553 | -0.055 | NA | NA | 9.15E-03 |
| GCST90274775 | CDCP1 | Hospitalized COVID-19 | WM | 34 | -0.069 (0.036) | 0.93 [0.87-1.00] | NA | NA | NA | NA | NA | 0.052 |
| GCST90274775 | CDCP1 | Hospitalized COVID-19 | MR-Egger | 34 | -0.041 (0.044) | 0.96 [0.88-1.05] | 30.96 | 0.519 | -0.066 | -0.003 | 0.582 | 0.355 |
| GCST90274776 | CSF-1 | Hospitalized COVID-19 | IVW | 29 | 0.000 (0.035) | 1.00 [0.93-1.07] | 36.59 | 0.128 | 0.235 | NA | NA | 0.999 |
| GCST90274776 | CSF-1 | Hospitalized COVID-19 | WM | 29 | -0.049 (0.048) | 0.95 [0.87-1.05] | NA | NA | NA | NA | NA | 0.303 |
| GCST90274776 | CSF-1 | Hospitalized COVID-19 | MR-Egger | 29 | -0.034 (0.083) | 0.97 [0.82-1.14] | 36.32 | 0.109 | 0.229 | 0.004 | 0.656 | 0.688 |
| GCST90274777 | CST5 | Hospitalized COVID-19 | IVW | 47 | 0.039 (0.023) | 1.04 [0.99-1.09] | 79.53 | 1.56E-03 | 0.422 | NA | NA | 0.093 |
| GCST90274777 | CST5 | Hospitalized COVID-19 | WM | 47 | 0.060 (0.026) | 1.06 [1.01-1.12] | NA | NA | NA | NA | NA | 0.022 |
| GCST90274777 | CST5 | Hospitalized COVID-19 | MR-Egger | 47 | 0.046 (0.037) | 1.05 [0.97-1.13] | 79.43 | 1.17E-03 | 0.421 | -0.001 | 0.813 | 0.224 |
| GCST90274778 | CX3CL1 | Hospitalized COVID-19 | IVW | 31 | 0.012 (0.058) | 1.01 [0.90-1.13] | 1.05E+02 | 2.71E-10 | 0.715 | NA | NA | 0.831 |
| GCST90274778 | CX3CL1 | Hospitalized COVID-19 | WM | 31 | 0.072 (0.045) | 1.07 [0.98-1.17] | NA | NA | NA | NA | NA | 0.114 |
| GCST90274778 | CX3CL1 | Hospitalized COVID-19 | MR-Egger | 31 | -0.137 (0.151) | 0.87 [0.65-1.17] | 1.01E+02 | 6.20E-10 | 0.704 | 0.014 | 0.293 | 0.371 |
| GCST90274779 | CXCL1 | Hospitalized COVID-19 | IVW | 26 | 0.052 (0.034) | 1.05 [0.98-1.13] | 40.39 | 0.027 | 0.381 | NA | NA | 0.129 |
| GCST90274779 | CXCL1 | Hospitalized COVID-19 | WM | 26 | 0.054 (0.035) | 1.06 [0.99-1.13] | NA | NA | NA | NA | NA | 0.122 |
| GCST90274779 | CXCL1 | Hospitalized COVID-19 | MR-Egger | 26 | 0.004 (0.055) | 1.00 [0.90-1.12] | 38.39 | 0.032 | 0.349 | 0.007 | 0.275 | 0.941 |
| GCST90274780 | CXCL10 | Hospitalized COVID-19 | IVW | 34 | 0.024 (0.034) | 1.02 [0.96-1.09] | 57.09 | 5.74E-03 | 0.422 | NA | NA | 0.482 |
| GCST90274780 | CXCL10 | Hospitalized COVID-19 | WM | 34 | 0.026 (0.039) | 1.03 [0.95-1.11] | NA | NA | NA | NA | NA | 0.51 |
| GCST90274780 | CXCL10 | Hospitalized COVID-19 | MR-Egger | 34 | 0.004 (0.061) | 1.00 [0.89-1.13] | 56.82 | 4.42E-03 | 0.419 | 0.002 | 0.699 | 0.949 |
| GCST90274781 | CXCL11 | Hospitalized COVID-19 | IVW | 38 | -0.052 (0.025) | 0.95 [0.90-1.00] | 27.62 | 0.869 | -0.34 | NA | NA | 0.037 |
| GCST90274781 | CXCL11 | Hospitalized COVID-19 | WM | 38 | -0.096 (0.038) | 0.91 [0.84-0.98] | NA | NA | NA | NA | NA | 0.012 |
| GCST90274781 | CXCL11 | Hospitalized COVID-19 | MR-Egger | 38 | -0.115 (0.057) | 0.89 [0.80-1.00] | 26.1 | 0.888 | -0.417 | 0.008 | 0.227 | 0.052 |
| GCST90274782 | CXCL5 | Hospitalized COVID-19 | IVW | 23 | 0.037 (0.023) | 1.04 [0.99-1.09] | 26.04 | 0.25 | 0.155 | NA | NA | 0.11 |
| GCST90274782 | CXCL5 | Hospitalized COVID-19 | WM | 23 | 0.018 (0.028) | 1.02 [0.96-1.08] | NA | NA | NA | NA | NA | 0.524 |
| GCST90274782 | CXCL5 | Hospitalized COVID-19 | MR-Egger | 23 | -0.003 (0.035) | 1.00 [0.93-1.07] | 23.55 | 0.315 | 0.066 | 0.007 | 0.151 | 0.925 |
| GCST90274783 | CXCL6 | Hospitalized COVID-19 | IVW | 24 | -0.015 (0.025) | 0.99 [0.94-1.03] | 29.59 | 0.162 | 0.223 | NA | NA | 0.552 |
| GCST90274783 | CXCL6 | Hospitalized COVID-19 | WM | 24 | -0.025 (0.025) | 0.98 [0.93-1.02] | NA | NA | NA | NA | NA | 0.322 |
| GCST90274783 | CXCL6 | Hospitalized COVID-19 | MR-Egger | 24 | -0.029 (0.038) | 0.97 [0.90-1.05] | 29.25 | 0.138 | 0.214 | 0.003 | 0.621 | 0.454 |
| GCST90274784 | CXCL9 | Hospitalized COVID-19 | IVW | 38 | -0.059 (0.042) | 0.94 [0.87-1.02] | 80.63 | 4.43E-05 | 0.541 | NA | NA | 0.155 |
| GCST90274784 | CXCL9 | Hospitalized COVID-19 | WM | 38 | -0.049 (0.043) | 0.95 [0.87-1.04] | NA | NA | NA | NA | NA | 0.259 |
| GCST90274784 | CXCL9 | Hospitalized COVID-19 | MR-Egger | 38 | -0.060 (0.095) | 0.94 [0.78-1.13] | 80.63 | 2.88E-05 | 0.541 | 0 | 0.993 | 0.532 |
| GCST90274785 | DNER | Hospitalized COVID-19 | IVW | 27 | 0.026 (0.045) | 1.03 [0.94-1.12] | 65.72 | 2.73E-05 | 0.604 | NA | NA | 0.561 |
| GCST90274785 | DNER | Hospitalized COVID-19 | WM | 27 | 0.035 (0.044) | 1.04 [0.95-1.13] | NA | NA | NA | NA | NA | 0.427 |
| GCST90274785 | DNER | Hospitalized COVID-19 | MR-Egger | 27 | 0.101 (0.096) | 1.11 [0.92-1.34] | 63.74 | 3.13E-05 | 0.592 | -0.008 | 0.386 | 0.303 |
| GCST90274786 | EN-RAGE | Hospitalized COVID-19 | IVW | 27 | 0.019 (0.032) | 1.02 [0.96-1.08] | 18.13 | 0.871 | -0.434 | NA | NA | 0.553 |
| GCST90274786 | EN-RAGE | Hospitalized COVID-19 | WM | 27 | 0.018 (0.048) | 1.02 [0.93-1.12] | NA | NA | NA | NA | NA | 0.711 |
| GCST90274786 | EN-RAGE | Hospitalized COVID-19 | MR-Egger | 27 | 0.011 (0.068) | 1.01 [0.88-1.16] | 18.11 | 0.838 | -0.436 | 0.001 | 0.899 | 0.872 |
| GCST90274787 | FGF-19 | Hospitalized COVID-19 | IVW | 33 | 0.082 (0.036) | 1.09 [1.01-1.17] | 57.52 | 3.70E-03 | 0.444 | NA | NA | 0.025 |
| GCST90274787 | FGF-19 | Hospitalized COVID-19 | WM | 33 | 0.042 (0.047) | 1.04 [0.95-1.14] | NA | NA | NA | NA | NA | 0.365 |
| GCST90274787 | FGF-19 | Hospitalized COVID-19 | MR-Egger | 33 | 0.269 (0.086) | 1.31 [1.11-1.55] | 48.59 | 0.023 | 0.341 | -0.023 | 0.023 | 3.65E-03 |
| GCST90274788 | FGF-21 | Hospitalized COVID-19 | IVW | 26 | -0.023 (0.030) | 0.98 [0.92-1.04] | 27.44 | 0.334 | 0.089 | NA | NA | 0.437 |
| GCST90274788 | FGF-21 | Hospitalized COVID-19 | WM | 26 | -0.027 (0.046) | 0.97 [0.89-1.07] | NA | NA | NA | NA | NA | 0.558 |
| GCST90274788 | FGF-21 | Hospitalized COVID-19 | MR-Egger | 26 | -0.043 (0.065) | 0.96 [0.84-1.09] | 27.31 | 0.29 | 0.085 | 0.002 | 0.739 | 0.522 |
| GCST90274789 | FGF-23 | Hospitalized COVID-19 | IVW | 26 | -0.038 (0.035) | 0.96 [0.90-1.03] | 17.32 | 0.87 | -0.443 | NA | NA | 0.279 |
| GCST90274789 | FGF-23 | Hospitalized COVID-19 | WM | 26 | -0.041 (0.048) | 0.96 [0.87-1.06] | NA | NA | NA | NA | NA | 0.4 |
| GCST90274789 | FGF-23 | Hospitalized COVID-19 | MR-Egger | 26 | 0.051 (0.089) | 1.05 [0.88-1.25] | 16.15 | 0.883 | -0.548 | -0.008 | 0.289 | 0.57 |
| GCST90274790 | FGF-5 | Hospitalized COVID-19 | IVW | 34 | -0.016 (0.023) | 0.98 [0.94-1.03] | 41.24 | 0.154 | 0.2 | NA | NA | 0.471 |
| GCST90274790 | FGF-5 | Hospitalized COVID-19 | WM | 34 | -0.014 (0.027) | 0.99 [0.94-1.04] | NA | NA | NA | NA | NA | 0.615 |
| GCST90274790 | FGF-5 | Hospitalized COVID-19 | MR-Egger | 34 | -0.019 (0.035) | 0.98 [0.92-1.05] | 41.23 | 0.127 | 0.2 | 0 | 0.933 | 0.602 |
| GCST90274791 | FIt3L | Hospitalized COVID-19 | IVW | 46 | 0.006 (0.021) | 1.01 [0.97-1.05] | 42.45 | 0.58 | -0.06 | NA | NA | 0.776 |
| GCST90274791 | FIt3L | Hospitalized COVID-19 | WM | 46 | 0.008 (0.035) | 1.01 [0.94-1.08] | NA | NA | NA | NA | NA | 0.819 |
| GCST90274791 | FIt3L | Hospitalized COVID-19 | MR-Egger | 46 | -0.036 (0.036) | 0.96 [0.90-1.04] | 40.44 | 0.625 | -0.113 | 0.005 | 0.163 | 0.328 |
| GCST90274792 | hGDNF | Hospitalized COVID-19 | IVW | 25 | -0.063 (0.033) | 0.94 [0.88-1.00] | 33.83 | 0.088 | 0.291 | NA | NA | 0.055 |
| GCST90274792 | hGDNF | Hospitalized COVID-19 | WM | 25 | -0.053 (0.039) | 0.95 [0.88-1.02] | NA | NA | NA | NA | NA | 0.181 |
| GCST90274792 | hGDNF | Hospitalized COVID-19 | MR-Egger | 25 | -0.025 (0.057) | 0.98 [0.87-1.09] | 32.87 | 0.083 | 0.27 | -0.006 | 0.421 | 0.667 |
| GCST90274793 | HGF | Hospitalized COVID-19 | IVW | 31 | -0.018 (0.031) | 0.98 [0.92-1.04] | 30.16 | 0.457 | 0.00536 | NA | NA | 0.572 |
| GCST90274793 | HGF | Hospitalized COVID-19 | WM | 31 | -0.042 (0.045) | 0.96 [0.88-1.05] | NA | NA | NA | NA | NA | 0.347 |
| GCST90274793 | HGF | Hospitalized COVID-19 | MR-Egger | 31 | 0.055 (0.066) | 1.06 [0.93-1.20] | 28.6 | 0.486 | -0.049 | -0.009 | 0.221 | 0.409 |
| GCST90274794 | IFN-γ | Hospitalized COVID-19 | IVW | 19 | -0.081 (0.038) | 0.92 [0.86-0.99] | 15.71 | 0.613 | -0.146 | NA | NA | 0.032 |
| GCST90274794 | IFN-γ | Hospitalized COVID-19 | WM | 19 | -0.056 (0.052) | 0.95 [0.85-1.05] | NA | NA | NA | NA | NA | 0.289 |
| GCST90274794 | IFN-γ | Hospitalized COVID-19 | MR-Egger | 19 | -0.007 (0.071) | 0.99 [0.86-1.14] | 14.21 | 0.652 | -0.267 | -0.009 | 0.237 | 0.923 |
| GCST90274795 | IL-10 | Hospitalized COVID-19 | IVW | 32 | 0.052 (0.030) | 1.05 [0.99-1.12] | 29.6 | 0.538 | -0.047 | NA | NA | 0.083 |
| GCST90274795 | IL-10 | Hospitalized COVID-19 | WM | 32 | 0.030 (0.044) | 1.03 [0.94-1.12] | NA | NA | NA | NA | NA | 0.501 |
| GCST90274795 | IL-10 | Hospitalized COVID-19 | MR-Egger | 32 | -0.030 (0.064) | 0.97 [0.86-1.10] | 27.56 | 0.594 | -0.125 | 0.009 | 0.164 | 0.649 |
| GCST90274796 | IL-10RA | Hospitalized COVID-19 | IVW | 20 | -0.003 (0.038) | 1.00 [0.93-1.07] | 14.78 | 0.737 | -0.286 | NA | NA | 0.942 |
| GCST90274796 | IL-10RA | Hospitalized COVID-19 | WM | 20 | -0.032 (0.050) | 0.97 [0.88-1.07] | NA | NA | NA | NA | NA | 0.517 |
| GCST90274796 | IL-10RA | Hospitalized COVID-19 | MR-Egger | 20 | 0.039 (0.091) | 1.04 [0.87-1.24] | 14.52 | 0.695 | -0.309 | -0.004 | 0.616 | 0.669 |
| GCST90274797 | IL10RB | Hospitalized COVID-19 | IVW | 29 | 0.075 (0.023) | 1.08 [1.03-1.13] | 38.42 | 0.091 | 0.271 | NA | NA | 1.23E-03 |
| GCST90274797 | IL10RB | Hospitalized COVID-19 | WM | 29 | 0.085 (0.023) | 1.09 [1.04-1.14] | NA | NA | NA | NA | NA | 2.78E-04 |
| GCST90274797 | IL10RB | Hospitalized COVID-19 | MR-Egger | 29 | 0.074 (0.033) | 1.08 [1.01-1.15] | 38.42 | 0.072 | 0.271 | 0 | 0.953 | 0.036 |
| GCST90274798 | IL-12B | Hospitalized COVID-19 | IVW | 38 | -0.020 (0.017) | 0.98 [0.95-1.01] | 43.94 | 0.201 | 0.158 | NA | NA | 0.237 |
| GCST90274798 | IL-12B | Hospitalized COVID-19 | WM | 38 | -0.044 (0.022) | 0.96 [0.92-1.00] | NA | NA | NA | NA | NA | 0.046 |
| GCST90274798 | IL-12B | Hospitalized COVID-19 | MR-Egger | 38 | -0.012 (0.027) | 0.99 [0.94-1.04] | 43.73 | 0.176 | 0.154 | -0.002 | 0.68 | 0.671 |
| GCST90274799 | IL-13 | Hospitalized COVID-19 | IVW | 28 | 0.005 (0.032) | 1.01 [0.94-1.07] | 26.8 | 0.475 | -0.00741 | NA | NA | 0.869 |
| GCST90274799 | IL-13 | Hospitalized COVID-19 | WM | 28 | 0.003 (0.043) | 1.00 [0.92-1.09] | NA | NA | NA | NA | NA | 0.949 |
| GCST90274799 | IL-13 | Hospitalized COVID-19 | MR-Egger | 28 | -0.007 (0.080) | 0.99 [0.85-1.16] | 26.77 | 0.421 | -0.00851 | 0.001 | 0.868 | 0.931 |
| GCST90274800 | IL-15RA | Hospitalized COVID-19 | IVW | 23 | -0.006 (0.022) | 0.99 [0.95-1.04] | 28.76 | 0.152 | 0.235 | NA | NA | 0.79 |
| GCST90274800 | IL-15RA | Hospitalized COVID-19 | WM | 23 | 0.004 (0.024) | 1.00 [0.96-1.05] | NA | NA | NA | NA | NA | 0.882 |
| GCST90274800 | IL-15RA | Hospitalized COVID-19 | MR-Egger | 23 | 0.009 (0.037) | 1.01 [0.94-1.09] | 28.4 | 0.129 | 0.225 | -0.003 | 0.612 | 0.807 |
| GCST90274801 | IL-17A | Hospitalized COVID-19 | IVW | 22 | 0.026 (0.036) | 1.03 [0.96-1.10] | 21.94 | 0.403 | 0.043 | NA | NA | 0.461 |
| GCST90274801 | IL-17A | Hospitalized COVID-19 | WM | 22 | 0.018 (0.049) | 1.02 [0.92-1.12] | NA | NA | NA | NA | NA | 0.716 |
| GCST90274801 | IL-17A | Hospitalized COVID-19 | MR-Egger | 22 | -0.043 (0.079) | 0.96 [0.82-1.12] | 20.9 | 0.403 | -0.00489 | 0.008 | 0.33 | 0.587 |
| GCST90274802 | IL-17C | Hospitalized COVID-19 | IVW | 37 | -0.054 (0.030) | 0.95 [0.89-1.01] | 47.5 | 0.095 | 0.242 | NA | NA | 0.078 |
| GCST90274802 | IL-17C | Hospitalized COVID-19 | WM | 37 | -0.079 (0.039) | 0.92 [0.86-1.00] | NA | NA | NA | NA | NA | 0.044 |
| GCST90274802 | IL-17C | Hospitalized COVID-19 | MR-Egger | 37 | -0.060 (0.072) | 0.94 [0.82-1.09] | 47.49 | 0.077 | 0.242 | 0.001 | 0.928 | 0.414 |
| GCST90274803 | IL-18 | Hospitalized COVID-19 | IVW | 32 | -0.050 (0.026) | 0.95 [0.90-1.00] | 34 | 0.325 | 0.088 | NA | NA | 0.057 |
| GCST90274803 | IL-18 | Hospitalized COVID-19 | WM | 32 | -0.059 (0.042) | 0.94 [0.87-1.02] | NA | NA | NA | NA | NA | 0.157 |
| GCST90274803 | IL-18 | Hospitalized COVID-19 | MR-Egger | 32 | -0.133 (0.053) | 0.88 [0.79-0.97] | 30.66 | 0.432 | -0.011 | 0.011 | 0.081 | 0.017 |
| GCST90274804 | IL-18R1 | Hospitalized COVID-19 | IVW | 38 | -0.009 (0.016) | 0.99 [0.96-1.02] | 33.7 | 0.625 | -0.098 | NA | NA | 0.585 |
| GCST90274804 | IL-18R1 | Hospitalized COVID-19 | WM | 38 | -0.027 (0.020) | 0.97 [0.94-1.01] | NA | NA | NA | NA | NA | 0.183 |
| GCST90274804 | IL-18R1 | Hospitalized COVID-19 | MR-Egger | 38 | -0.029 (0.023) | 0.97 [0.93-1.02] | 32.28 | 0.646 | -0.146 | 0.005 | 0.242 | 0.223 |
| GCST90274805 | IL-1α | Hospitalized COVID-19 | IVW | 22 | -0.020 (0.032) | 0.98 [0.92-1.04] | 16.45 | 0.744 | -0.277 | NA | NA | 0.531 |
| GCST90274805 | IL-1α | Hospitalized COVID-19 | WM | 22 | 0.043 (0.046) | 1.04 [0.95-1.14] | NA | NA | NA | NA | NA | 0.353 |
| GCST90274805 | IL-1α | Hospitalized COVID-19 | MR-Egger | 22 | -0.006 (0.067) | 0.99 [0.87-1.13] | 16.39 | 0.692 | -0.281 | -0.002 | 0.813 | 0.925 |
| GCST90274806 | IL-2 | Hospitalized COVID-19 | IVW | 21 | 0.065 (0.037) | 1.07 [0.99-1.15] | 20.18 | 0.447 | 0.00885 | NA | NA | 0.075 |
| GCST90274806 | IL-2 | Hospitalized COVID-19 | WM | 21 | 0.059 (0.053) | 1.06 [0.96-1.18] | NA | NA | NA | NA | NA | 0.261 |
| GCST90274806 | IL-2 | Hospitalized COVID-19 | MR-Egger | 21 | 0.142 (0.092) | 1.15 [0.96-1.38] | 19.34 | 0.435 | -0.034 | -0.008 | 0.376 | 0.139 |
| GCST90274807 | IL-20 | Hospitalized COVID-19 | IVW | 23 | 0.051 (0.035) | 1.05 [0.98-1.13] | 17.59 | 0.73 | -0.251 | NA | NA | 0.147 |
| GCST90274807 | IL-20 | Hospitalized COVID-19 | WM | 23 | 0.032 (0.049) | 1.03 [0.94-1.14] | NA | NA | NA | NA | NA | 0.52 |
| GCST90274807 | IL-20 | Hospitalized COVID-19 | MR-Egger | 23 | -0.025 (0.075) | 0.98 [0.84-1.13] | 16.29 | 0.753 | -0.35 | 0.011 | 0.267 | 0.744 |
| GCST90274808 | IL-20RA | Hospitalized COVID-19 | IVW | 22 | 0.028 (0.035) | 1.03 [0.96-1.10] | 9.24 | 0.987 | -1.27 | NA | NA | 0.428 |
| GCST90274808 | IL-20RA | Hospitalized COVID-19 | WM | 22 | 0.014 (0.048) | 1.01 [0.92-1.11] | NA | NA | NA | NA | NA | 0.775 |
| GCST90274808 | IL-20RA | Hospitalized COVID-19 | MR-Egger | 22 | -0.017 (0.076) | 0.98 [0.85-1.14] | 8.8 | 0.985 | -1.39 | 0.006 | 0.515 | 0.828 |
| GCST90274809 | IL-22RA1 | Hospitalized COVID-19 | IVW | 21 | -0.037 (0.043) | 0.96 [0.89-1.05] | 29.07 | 0.086 | 0.312 | NA | NA | 0.397 |
| GCST90274809 | IL-22RA1 | Hospitalized COVID-19 | WM | 21 | -0.032 (0.052) | 0.97 [0.88-1.07] | NA | NA | NA | NA | NA | 0.541 |
| GCST90274809 | IL-22RA1 | Hospitalized COVID-19 | MR-Egger | 21 | -0.022 (0.085) | 0.98 [0.83-1.16] | 29 | 0.066 | 0.31 | -0.002 | 0.837 | 0.801 |
| GCST90274810 | IL-24 | Hospitalized COVID-19 | IVW | 19 | -0.038 (0.040) | 0.96 [0.89-1.04] | 20.76 | 0.292 | 0.133 | NA | NA | 0.339 |
| GCST90274810 | IL-24 | Hospitalized COVID-19 | WM | 19 | -0.095 (0.052) | 0.91 [0.82-1.01] | NA | NA | NA | NA | NA | 0.064 |
| GCST90274810 | IL-24 | Hospitalized COVID-19 | MR-Egger | 19 | 0.110 (0.078) | 1.12 [0.96-1.30] | 16.13 | 0.514 | -0.116 | -0.017 | 0.046 | 0.177 |
| GCST90274811 | IL-2RB | Hospitalized COVID-19 | IVW | 22 | -0.011 (0.036) | 0.99 [0.92-1.06] | 9.27 | 0.987 | -1.27 | NA | NA | 0.767 |
| GCST90274811 | IL-2RB | Hospitalized COVID-19 | WM | 22 | -0.016 (0.049) | 0.98 [0.89-1.08] | NA | NA | NA | NA | NA | 0.742 |
| GCST90274811 | IL-2RB | Hospitalized COVID-19 | MR-Egger | 22 | -0.125 (0.074) | 0.88 [0.76-1.02] | 6.19 | 0.999 | -2.39 | 0.013 | 0.095 | 0.109 |
| GCST90274812 | IL-33 | Hospitalized COVID-19 | IVW | 22 | 0.003 (0.041) | 1.00 [0.93-1.09] | 26.72 | 0.18 | 0.214 | NA | NA | 0.947 |
| GCST90274812 | IL-33 | Hospitalized COVID-19 | WM | 22 | 0.008 (0.053) | 1.01 [0.91-1.12] | NA | NA | NA | NA | NA | 0.879 |
| GCST90274812 | IL-33 | Hospitalized COVID-19 | MR-Egger | 22 | 0.166 (0.101) | 1.18 [0.97-1.44] | 23.16 | 0.281 | 0.093 | -0.017 | 0.095 | 0.116 |
| GCST90274813 | IL-4 | Hospitalized COVID-19 | IVW | 22 | -0.010 (0.036) | 0.99 [0.92-1.06] | 18.29 | 0.631 | -0.148 | NA | NA | 0.778 |
| GCST90274813 | IL-4 | Hospitalized COVID-19 | WM | 22 | -0.038 (0.049) | 0.96 [0.87-1.06] | NA | NA | NA | NA | NA | 0.445 |
| GCST90274813 | IL-4 | Hospitalized COVID-19 | MR-Egger | 22 | -0.086 (0.092) | 0.92 [0.77-1.10] | 17.5 | 0.621 | -0.2 | 0.007 | 0.384 | 0.363 |
| GCST90274814 | IL-5 | Hospitalized COVID-19 | IVW | 22 | -0.000 (0.035) | 1.00 [0.93-1.07] | 15.16 | 0.815 | -0.386 | NA | NA | 0.993 |
| GCST90274814 | IL-5 | Hospitalized COVID-19 | WM | 22 | 0.027 (0.048) | 1.03 [0.94-1.13] | NA | NA | NA | NA | NA | 0.569 |
| GCST90274814 | IL-5 | Hospitalized COVID-19 | MR-Egger | 22 | 0.045 (0.083) | 1.05 [0.89-1.23] | 14.8 | 0.788 | -0.419 | -0.005 | 0.555 | 0.594 |
| GCST90274815 | IL-6 | Hospitalized COVID-19 | IVW | 14 | -0.063 (0.039) | 0.94 [0.87-1.01] | 13.42 | 0.416 | 0.032 | NA | NA | 0.104 |
| GCST90274815 | IL-6 | Hospitalized COVID-19 | WM | 14 | -0.115 (0.053) | 0.89 [0.80-0.99] | NA | NA | NA | NA | NA | 0.029 |
| GCST90274815 | IL-6 | Hospitalized COVID-19 | MR-Egger | 14 | -0.036 (0.080) | 0.96 [0.82-1.13] | 13.25 | 0.351 | 0.019 | -0.004 | 0.7 | 0.66 |
| GCST90274816 | IL-7 | Hospitalized COVID-19 | IVW | 23 | 0.064 (0.049) | 1.07 [0.97-1.17] | 41.82 | 6.57E-03 | 0.474 | NA | NA | 0.187 |
| GCST90274816 | IL-7 | Hospitalized COVID-19 | WM | 23 | 0.017 (0.055) | 1.02 [0.91-1.13] | NA | NA | NA | NA | NA | 0.761 |
| GCST90274816 | IL-7 | Hospitalized COVID-19 | MR-Egger | 23 | -0.096 (0.116) | 0.91 [0.72-1.14] | 37.7 | 0.014 | 0.417 | 0.017 | 0.145 | 0.416 |
| GCST90274817 | IL-8 | Hospitalized COVID-19 | IVW | 29 | -0.019 (0.033) | 0.98 [0.92-1.05] | 26.78 | 0.53 | -0.046 | NA | NA | 0.569 |
| GCST90274817 | IL-8 | Hospitalized COVID-19 | WM | 29 | -0.008 (0.048) | 0.99 [0.90-1.09] | NA | NA | NA | NA | NA | 0.875 |
| GCST90274817 | IL-8 | Hospitalized COVID-19 | MR-Egger | 29 | 0.028 (0.069) | 1.03 [0.90-1.18] | 26.16 | 0.51 | -0.07 | -0.005 | 0.441 | 0.685 |
| GCST90274818 | LAP TGF-β-1 | Hospitalized COVID-19 | IVW | 29 | 0.005 (0.031) | 1.01 [0.95-1.07] | 26.19 | 0.562 | -0.069 | NA | NA | 0.863 |
| GCST90274818 | LAP TGF-β-1 | Hospitalized COVID-19 | WM | 29 | -0.019 (0.044) | 0.98 [0.90-1.07] | NA | NA | NA | NA | NA | 0.673 |
| GCST90274818 | LAP TGF-β-1 | Hospitalized COVID-19 | MR-Egger | 29 | -0.091 (0.054) | 0.91 [0.82-1.01] | 21.46 | 0.764 | -0.305 | 0.011 | 0.039 | 0.102 |
| GCST90274819 | LIF | Hospitalized COVID-19 | IVW | 27 | 0.066 (0.036) | 1.07 [1.00-1.15] | 33.23 | 0.156 | 0.217 | NA | NA | 0.065 |
| GCST90274819 | LIF | Hospitalized COVID-19 | WM | 27 | 0.037 (0.048) | 1.04 [0.94-1.14] | NA | NA | NA | NA | NA | 0.449 |
| GCST90274819 | LIF | Hospitalized COVID-19 | MR-Egger | 27 | 0.110 (0.077) | 1.12 [0.96-1.30] | 32.68 | 0.139 | 0.205 | -0.005 | 0.526 | 0.166 |
| GCST90274820 | LIF-R | Hospitalized COVID-19 | IVW | 29 | -0.124 (0.037) | 0.88 [0.82-0.95] | 59.04 | 5.39E-04 | 0.526 | NA | NA | 7.50E-04 |
| GCST90274820 | LIF-R | Hospitalized COVID-19 | WM | 29 | -0.111 (0.051) | 0.90 [0.81-0.99] | NA | NA | NA | NA | NA | 0.032 |
| GCST90274820 | LIF-R | Hospitalized COVID-19 | MR-Egger | 29 | -0.299 (0.052) | 0.74 [0.67-0.82] | 36.79 | 0.099 | 0.239 | 0.022 | 3.97E-04 | 4.64E-06 |
| GCST90274821 | CCL2 | Hospitalized COVID-19 | IVW | 30 | 0.064 (0.030) | 1.07 [1.00-1.13] | 39.2 | 0.098 | 0.26 | NA | NA | 0.034 |
| GCST90274821 | CCL2 | Hospitalized COVID-19 | WM | 30 | 0.056 (0.042) | 1.06 [0.97-1.15] | NA | NA | NA | NA | NA | 0.19 |
| GCST90274821 | CCL2 | Hospitalized COVID-19 | MR-Egger | 30 | 0.120 (0.058) | 1.13 [1.01-1.26] | 37.47 | 0.109 | 0.226 | -0.007 | 0.265 | 0.047 |
| GCST90274822 | CCL8 | Hospitalized COVID-19 | IVW | 45 | -0.008 (0.016) | 0.99 [0.96-1.02] | 57.12 | 0.089 | 0.23 | NA | NA | 0.599 |
| GCST90274822 | CCL8 | Hospitalized COVID-19 | WM | 45 | 0.030 (0.022) | 1.03 [0.99-1.08] | NA | NA | NA | NA | NA | 0.172 |
| GCST90274822 | CCL8 | Hospitalized COVID-19 | MR-Egger | 45 | 0.021 (0.019) | 1.02 [0.99-1.06] | 49.29 | 0.236 | 0.107 | -0.01 | 0.012 | 0.256 |
| GCST90274823 | CCL7 | Hospitalized COVID-19 | IVW | 27 | -0.024 (0.028) | 0.98 [0.92-1.03] | 20.45 | 0.77 | -0.272 | NA | NA | 0.379 |
| GCST90274823 | CCL7 | Hospitalized COVID-19 | WM | 27 | -0.016 (0.043) | 0.98 [0.90-1.07] | NA | NA | NA | NA | NA | 0.715 |
| GCST90274823 | CCL7 | Hospitalized COVID-19 | MR-Egger | 27 | -0.028 (0.062) | 0.97 [0.86-1.10] | 20.44 | 0.723 | -0.272 | 0 | 0.953 | 0.662 |
| GCST90274824 | CCL13 | Hospitalized COVID-19 | IVW | 30 | -0.018 (0.023) | 0.98 [0.94-1.03] | 30.01 | 0.413 | 0.034 | NA | NA | 0.439 |
| GCST90274824 | CCL13 | Hospitalized COVID-19 | WM | 30 | -0.027 (0.033) | 0.97 [0.91-1.04] | NA | NA | NA | NA | NA | 0.419 |
| GCST90274824 | CCL13 | Hospitalized COVID-19 | MR-Egger | 30 | -0.036 (0.046) | 0.96 [0.88-1.06] | 29.79 | 0.374 | 0.026 | 0.003 | 0.647 | 0.44 |
| GCST90274825 | MIP-1α | Hospitalized COVID-19 | IVW | 21 | -0.020 (0.030) | 0.98 [0.92-1.04] | 35.6 | 0.017 | 0.438 | NA | NA | 0.515 |
| GCST90274825 | MIP-1α | Hospitalized COVID-19 | WM | 21 | -0.014 (0.028) | 0.99 [0.93-1.04] | NA | NA | NA | NA | NA | 0.616 |
| GCST90274825 | MIP-1α | Hospitalized COVID-19 | MR-Egger | 21 | 0.001 (0.047) | 1.00 [0.91-1.10] | 35 | 0.014 | 0.429 | -0.004 | 0.574 | 0.984 |
| GCST90274826 | MMP-1 | Hospitalized COVID-19 | IVW | 26 | 0.002 (0.037) | 1.00 [0.93-1.08] | 42.25 | 0.017 | 0.408 | NA | NA | 0.951 |
| GCST90274826 | MMP-1 | Hospitalized COVID-19 | WM | 26 | -0.007 (0.045) | 0.99 [0.91-1.08] | NA | NA | NA | NA | NA | 0.884 |
| GCST90274826 | MMP-1 | Hospitalized COVID-19 | MR-Egger | 26 | 0.044 (0.071) | 1.05 [0.91-1.20] | 41.4 | 0.015 | 0.396 | -0.005 | 0.491 | 0.537 |
| GCST90274827 | MMP-10 | Hospitalized COVID-19 | IVW | 24 | -0.019 (0.021) | 0.98 [0.94-1.02] | 24.84 | 0.358 | 0.074 | NA | NA | 0.37 |
| GCST90274827 | MMP-10 | Hospitalized COVID-19 | WM | 24 | -0.027 (0.027) | 0.97 [0.92-1.03] | NA | NA | NA | NA | NA | 0.318 |
| GCST90274827 | MMP-10 | Hospitalized COVID-19 | MR-Egger | 24 | -0.042 (0.028) | 0.96 [0.91-1.01] | 23.23 | 0.389 | 0.00993 | 0.006 | 0.23 | 0.149 |
| GCST90274828 | NRTN | Hospitalized COVID-19 | IVW | 23 | 0.022 (0.045) | 1.02 [0.94-1.12] | 35.91 | 0.031 | 0.387 | NA | NA | 0.632 |
| GCST90274828 | NRTN | Hospitalized COVID-19 | WM | 23 | 0.051 (0.054) | 1.05 [0.95-1.17] | NA | NA | NA | NA | NA | 0.347 |
| GCST90274828 | NRTN | Hospitalized COVID-19 | MR-Egger | 23 | -0.057 (0.094) | 0.94 [0.78-1.14] | 34.42 | 0.033 | 0.361 | 0.008 | 0.352 | 0.552 |
| GCST90274829 | NT-3 | Hospitalized COVID-19 | IVW | 30 | 0.052 (0.040) | 1.05 [0.97-1.14] | 42.9 | 0.046 | 0.324 | NA | NA | 0.198 |
| GCST90274829 | NT-3 | Hospitalized COVID-19 | WM | 30 | 0.060 (0.050) | 1.06 [0.96-1.17] | NA | NA | NA | NA | NA | 0.234 |
| GCST90274829 | NT-3 | Hospitalized COVID-19 | MR-Egger | 30 | 0.113 (0.089) | 1.12 [0.94-1.33] | 42.02 | 0.043 | 0.31 | -0.006 | 0.449 | 0.216 |
| GCST90274830 | OPG | Hospitalized COVID-19 | IVW | 31 | -0.002 (0.027) | 1.00 [0.95-1.05] | 28.69 | 0.534 | -0.046 | NA | NA | 0.954 |
| GCST90274830 | OPG | Hospitalized COVID-19 | WM | 31 | -0.005 (0.043) | 0.99 [0.92-1.08] | NA | NA | NA | NA | NA | 0.9 |
| GCST90274830 | OPG | Hospitalized COVID-19 | MR-Egger | 31 | -0.005 (0.056) | 1.00 [0.89-1.11] | 28.69 | 0.482 | -0.046 | 0 | 0.951 | 0.935 |
| GCST90274831 | OSM | Hospitalized COVID-19 | IVW | 24 | -0.043 (0.045) | 0.96 [0.88-1.05] | 42.28 | 8.43E-03 | 0.456 | NA | NA | 0.34 |
| GCST90274831 | OSM | Hospitalized COVID-19 | WM | 24 | -0.027 (0.051) | 0.97 [0.88-1.08] | NA | NA | NA | NA | NA | 0.604 |
| GCST90274831 | OSM | Hospitalized COVID-19 | MR-Egger | 24 | 0.015 (0.120) | 1.02 [0.80-1.28] | 41.74 | 6.71E-03 | 0.449 | -0.006 | 0.601 | 0.899 |
| GCST90274832 | PD-L1 | Hospitalized COVID-19 | IVW | 31 | -0.058 (0.034) | 0.94 [0.88-1.01] | 32.96 | 0.324 | 0.09 | NA | NA | 0.082 |
| GCST90274832 | PD-L1 | Hospitalized COVID-19 | WM | 31 | -0.026 (0.049) | 0.97 [0.88-1.07] | NA | NA | NA | NA | NA | 0.596 |
| GCST90274832 | PD-L1 | Hospitalized COVID-19 | MR-Egger | 31 | -0.049 (0.076) | 0.95 [0.82-1.10] | 32.94 | 0.28 | 0.089 | -0.001 | 0.894 | 0.52 |
| GCST90274833 | SCF | Hospitalized COVID-19 | IVW | 46 | -0.013 (0.025) | 0.99 [0.94-1.04] | 65.97 | 0.022 | 0.318 | NA | NA | 0.606 |
| GCST90274833 | SCF | Hospitalized COVID-19 | WM | 46 | -0.033 (0.034) | 0.97 [0.91-1.03] | NA | NA | NA | NA | NA | 0.325 |
| GCST90274833 | SCF | Hospitalized COVID-19 | MR-Egger | 46 | -0.020 (0.050) | 0.98 [0.89-1.08] | 65.92 | 0.018 | 0.317 | 0.001 | 0.867 | 0.689 |
| GCST90274834 | SIRT2 | Hospitalized COVID-19 | IVW | 19 | 0.031 (0.046) | 1.03 [0.94-1.13] | 23.35 | 0.178 | 0.229 | NA | NA | 0.498 |
| GCST90274834 | SIRT2 | Hospitalized COVID-19 | WM | 19 | 0.077 (0.061) | 1.08 [0.96-1.22] | NA | NA | NA | NA | NA | 0.205 |
| GCST90274834 | SIRT2 | Hospitalized COVID-19 | MR-Egger | 19 | -0.003 (0.099) | 1.00 [0.82-1.21] | 23.14 | 0.145 | 0.222 | 0.004 | 0.7 | 0.976 |
| GCST90274835 | SLAMF1 | Hospitalized COVID-19 | IVW | 39 | -0.019 (0.025) | 0.98 [0.94-1.03] | 34.45 | 0.634 | -0.103 | NA | NA | 0.455 |
| GCST90274835 | SLAMF1 | Hospitalized COVID-19 | WM | 39 | 0.000 (0.038) | 1.00 [0.93-1.08] | NA | NA | NA | NA | NA | 0.99 |
| GCST90274835 | SLAMF1 | Hospitalized COVID-19 | MR-Egger | 39 | -0.001 (0.055) | 1.00 [0.90-1.11] | 34.32 | 0.595 | -0.107 | -0.002 | 0.716 | 0.992 |
| GCST90274836 | ST1A1 | Hospitalized COVID-19 | IVW | 33 | 0.023 (0.025) | 1.02 [0.97-1.07] | 27.08 | 0.714 | -0.182 | NA | NA | 0.369 |
| GCST90274836 | ST1A1 | Hospitalized COVID-19 | WM | 33 | 0.021 (0.035) | 1.02 [0.95-1.09] | NA | NA | NA | NA | NA | 0.546 |
| GCST90274836 | ST1A1 | Hospitalized COVID-19 | MR-Egger | 33 | 0.072 (0.060) | 1.08 [0.96-1.21] | 26.26 | 0.709 | -0.219 | -0.006 | 0.371 | 0.239 |
| GCST90274837 | STAMPB | Hospitalized COVID-19 | IVW | 21 | 0.033 (0.046) | 1.03 [0.95-1.13] | 25.93 | 0.168 | 0.229 | NA | NA | 0.468 |
| GCST90274837 | STAMPB | Hospitalized COVID-19 | WM | 21 | 0.013 (0.059) | 1.01 [0.90-1.14] | NA | NA | NA | NA | NA | 0.829 |
| GCST90274837 | STAMPB | Hospitalized COVID-19 | MR-Egger | 21 | 0.016 (0.110) | 1.02 [0.82-1.26] | 25.9 | 0.133 | 0.228 | 0.002 | 0.868 | 0.885 |
| GCST90274838 | TGF-α | Hospitalized COVID-19 | IVW | 27 | -0.003 (0.041) | 1.00 [0.92-1.08] | 40.77 | 0.033 | 0.362 | NA | NA | 0.94 |
| GCST90274838 | TGF-α | Hospitalized COVID-19 | WM | 27 | 0.029 (0.050) | 1.03 [0.93-1.13] | NA | NA | NA | NA | NA | 0.556 |
| GCST90274838 | TGF-α | Hospitalized COVID-19 | MR-Egger | 27 | 0.166 (0.091) | 1.18 [0.99-1.41] | 34.92 | 0.09 | 0.255 | -0.018 | 0.051 | 0.081 |
| GCST90274839 | TNF | Hospitalized COVID-19 | IVW | 29 | 0.003 (0.030) | 1.00 [0.94-1.06] | 23.28 | 0.719 | -0.203 | NA | NA | 0.933 |
| GCST90274839 | TNF | Hospitalized COVID-19 | WM | 29 | -0.011 (0.042) | 0.99 [0.91-1.07] | NA | NA | NA | NA | NA | 0.795 |
| GCST90274839 | TNF | Hospitalized COVID-19 | MR-Egger | 29 | 0.007 (0.063) | 1.01 [0.89-1.14] | 23.27 | 0.67 | -0.203 | 0 | 0.937 | 0.912 |
| GCST90274840 | TNFB | Hospitalized COVID-19 | IVW | 41 | 0.019 (0.018) | 1.02 [0.98-1.06] | 46.61 | 0.219 | 0.142 | NA | NA | 0.306 |
| GCST90274840 | TNFB | Hospitalized COVID-19 | WM | 41 | 0.048 (0.024) | 1.05 [1.00-1.10] | NA | NA | NA | NA | NA | 0.045 |
| GCST90274840 | TNFB | Hospitalized COVID-19 | MR-Egger | 41 | 0.014 (0.029) | 1.01 [0.96-1.07] | 46.56 | 0.189 | 0.141 | 0.001 | 0.838 | 0.635 |
| GCST90274841 | TNFRSF9 | Hospitalized COVID-19 | IVW | 34 | -0.017 (0.026) | 0.98 [0.93-1.04] | 22.78 | 0.909 | -0.449 | NA | NA | 0.521 |
| GCST90274841 | TNFRSF9 | Hospitalized COVID-19 | WM | 34 | 0.006 (0.038) | 1.01 [0.93-1.09] | NA | NA | NA | NA | NA | 0.866 |
| GCST90274841 | TNFRSF9 | Hospitalized COVID-19 | MR-Egger | 34 | 0.048 (0.058) | 1.05 [0.94-1.18] | 21.2 | 0.927 | -0.556 | -0.008 | 0.218 | 0.412 |
| GCST90274842 | TNFSF14 | Hospitalized COVID-19 | IVW | 39 | -0.011 (0.023) | 0.99 [0.95-1.03] | 40.49 | 0.361 | 0.061 | NA | NA | 0.643 |
| GCST90274842 | TNFSF14 | Hospitalized COVID-19 | WM | 39 | -0.012 (0.037) | 0.99 [0.92-1.06] | NA | NA | NA | NA | NA | 0.753 |
| GCST90274842 | TNFSF14 | Hospitalized COVID-19 | MR-Egger | 39 | -0.013 (0.037) | 0.99 [0.92-1.06] | 40.48 | 0.319 | 0.061 | 0 | 0.939 | 0.732 |
| GCST90274843 | TRAIL | Hospitalized COVID-19 | IVW | 37 | -0.000 (0.020) | 1.00 [0.96-1.04] | 39.57 | 0.313 | 0.09 | NA | NA | 0.983 |
| GCST90274843 | TRAIL | Hospitalized COVID-19 | WM | 37 | 0.036 (0.031) | 1.04 [0.98-1.10] | NA | NA | NA | NA | NA | 0.243 |
| GCST90274843 | TRAIL | Hospitalized COVID-19 | MR-Egger | 37 | -0.017 (0.033) | 0.98 [0.92-1.05] | 39.14 | 0.289 | 0.08 | 0.003 | 0.537 | 0.618 |
| GCST90274844 | TRANCE | Hospitalized COVID-19 | IVW | 49 | 0.019 (0.023) | 1.02 [0.97-1.07] | 56.82 | 0.179 | 0.155 | NA | NA | 0.412 |
| GCST90274844 | TRANCE | Hospitalized COVID-19 | WM | 49 | -0.003 (0.036) | 1.00 [0.93-1.07] | NA | NA | NA | NA | NA | 0.938 |
| GCST90274844 | TRANCE | Hospitalized COVID-19 | MR-Egger | 49 | -0.024 (0.047) | 0.98 [0.89-1.07] | 55.52 | 0.185 | 0.135 | 0.005 | 0.298 | 0.611 |
| GCST90274845 | TSLP | Hospitalized COVID-19 | IVW | 24 | -0.016 (0.034) | 0.98 [0.92-1.05] | 18.33 | 0.739 | -0.255 | NA | NA | 0.644 |
| GCST90274845 | TSLP | Hospitalized COVID-19 | WM | 24 | -0.015 (0.048) | 0.98 [0.90-1.08] | NA | NA | NA | NA | NA | 0.751 |
| GCST90274845 | TSLP | Hospitalized COVID-19 | MR-Egger | 24 | -0.025 (0.075) | 0.98 [0.84-1.13] | 18.31 | 0.688 | -0.256 | 0.001 | 0.887 | 0.739 |
| GCST90274846 | TWEAK | Hospitalized COVID-19 | IVW | 44 | -0.011 (0.040) | 0.99 [0.91-1.07] | 1.32E+02 | 6.62E-11 | 0.673 | NA | NA | 0.792 |
| GCST90274846 | TWEAK | Hospitalized COVID-19 | WM | 44 | 0.010 (0.042) | 1.01 [0.93-1.10] | NA | NA | NA | NA | NA | 0.819 |
| GCST90274846 | TWEAK | Hospitalized COVID-19 | MR-Egger | 44 | 0.067 (0.084) | 1.07 [0.91-1.26] | 1.28E+02 | 1.21E-10 | 0.664 | -0.009 | 0.297 | 0.427 |
| GCST90274847 | uPA | Hospitalized COVID-19 | IVW | 44 | 0.004 (0.026) | 1.00 [0.95-1.06] | 55.26 | 0.1 | 0.222 | NA | NA | 0.873 |
| GCST90274847 | uPA | Hospitalized COVID-19 | WM | 44 | -0.009 (0.035) | 0.99 [0.92-1.06] | NA | NA | NA | NA | NA | 0.794 |
| GCST90274847 | uPA | Hospitalized COVID-19 | MR-Egger | 44 | -0.080 (0.057) | 0.92 [0.82-1.03] | 51.89 | 0.141 | 0.171 | 0.009 | 0.106 | 0.169 |
| GCST90274848 | VEGF_A | Hospitalized COVID-19 | IVW | 34 | 0.022 (0.021) | 1.02 [0.98-1.06] | 31.6 | 0.537 | -0.044 | NA | NA | 0.284 |
| GCST90274848 | VEGF_A | Hospitalized COVID-19 | WM | 34 | 0.033 (0.027) | 1.03 [0.98-1.09] | NA | NA | NA | NA | NA | 0.229 |
| GCST90274848 | VEGF_A | Hospitalized COVID-19 | MR-Egger | 34 | 0.042 (0.035) | 1.04 [0.97-1.12] | 31.08 | 0.513 | -0.062 | -0.003 | 0.475 | 0.233 |
| GCST90274758 | 4EBP1 | Critical COVID-19 | IVW | 17 | 0.153 (0.072) | 1.16 [1.01-1.34] | 27.73 | 0.034 | 0.423 | NA | NA | 0.033 |
| GCST90274758 | 4EBP1 | Critical COVID-19 | WM | 17 | 0.080 (0.078) | 1.08 [0.93-1.26] | NA | NA | NA | NA | NA | 0.304 |
| GCST90274758 | 4EBP1 | Critical COVID-19 | MR-Egger | 17 | 0.241 (0.178) | 1.27 [0.90-1.80] | 27.2 | 0.027 | 0.412 | -0.009 | 0.595 | 0.196 |
| GCST90274759 | ADA | Critical COVID-19 | IVW | 24 | 0.010 (0.030) | 1.01 [0.95-1.07] | 28.82 | 0.186 | 0.202 | NA | NA | 0.737 |
| GCST90274759 | ADA | Critical COVID-19 | WM | 24 | 0.024 (0.033) | 1.02 [0.96-1.09] | NA | NA | NA | NA | NA | 0.46 |
| GCST90274759 | ADA | Critical COVID-19 | MR-Egger | 24 | 0.031 (0.038) | 1.03 [0.96-1.11] | 27.73 | 0.185 | 0.171 | -0.007 | 0.364 | 0.424 |
| GCST90274760 | ARTN | Critical COVID-19 | IVW | 31 | -0.049 (0.060) | 0.95 [0.85-1.07] | 54.54 | 4.00E-03 | 0.45 | NA | NA | 0.421 |
| GCST90274760 | ARTN | Critical COVID-19 | WM | 31 | -0.011 (0.070) | 0.99 [0.86-1.14] | NA | NA | NA | NA | NA | 0.881 |
| GCST90274760 | ARTN | Critical COVID-19 | MR-Egger | 31 | -0.120 (0.140) | 0.89 [0.67-1.17] | 53.94 | 3.28E-03 | 0.444 | 0.008 | 0.576 | 0.399 |
| GCST90274761 | AXIN1 | Critical COVID-19 | IVW | 13 | 0.106 (0.067) | 1.11 [0.97-1.27] | 11.84 | 0.458 | -0.013 | NA | NA | 0.115 |
| GCST90274761 | AXIN1 | Critical COVID-19 | WM | 13 | 0.085 (0.093) | 1.09 [0.91-1.31] | NA | NA | NA | NA | NA | 0.364 |
| GCST90274761 | AXIN1 | Critical COVID-19 | MR-Egger | 13 | 0.193 (0.176) | 1.21 [0.86-1.71] | 11.54 | 0.399 | -0.04 | -0.009 | 0.602 | 0.296 |
| GCST90274762 | β-NGF | Critical COVID-19 | IVW | 32 | 0.002 (0.045) | 1.00 [0.92-1.10] | 22.31 | 0.873 | -0.39 | NA | NA | 0.968 |
| GCST90274762 | β-NGF | Critical COVID-19 | WM | 32 | 0.003 (0.063) | 1.00 [0.89-1.13] | NA | NA | NA | NA | NA | 0.961 |
| GCST90274762 | β-NGF | Critical COVID-19 | MR-Egger | 32 | 0.015 (0.099) | 1.01 [0.84-1.23] | 22.29 | 0.843 | -0.391 | -0.001 | 0.884 | 0.883 |
| GCST90274763 | CASP-8 | Critical COVID-19 | IVW | 22 | 0.064 (0.065) | 1.07 [0.94-1.21] | 34.63 | 0.031 | 0.394 | NA | NA | 0.322 |
| GCST90274763 | CASP-8 | Critical COVID-19 | WM | 22 | 0.116 (0.077) | 1.12 [0.97-1.31] | NA | NA | NA | NA | NA | 0.132 |
| GCST90274763 | CASP-8 | Critical COVID-19 | MR-Egger | 22 | 0.204 (0.149) | 1.23 [0.92-1.64] | 32.83 | 0.035 | 0.36 | -0.015 | 0.308 | 0.184 |
| GCST90274764 | CCL11 | Critical COVID-19 | IVW | 29 | -0.012 (0.048) | 0.99 [0.90-1.09] | 40.35 | 0.062 | 0.306 | NA | NA | 0.807 |
| GCST90274764 | CCL11 | Critical COVID-19 | WM | 29 | -0.057 (0.057) | 0.94 [0.84-1.06] | NA | NA | NA | NA | NA | 0.318 |
| GCST90274764 | CCL11 | Critical COVID-19 | MR-Egger | 29 | -0.086 (0.095) | 0.92 [0.76-1.11] | 39.16 | 0.061 | 0.285 | 0.01 | 0.373 | 0.373 |
| GCST90274765 | CCL19 | Critical COVID-19 | IVW | 37 | -0.005 (0.037) | 0.99 [0.92-1.07] | 36.09 | 0.464 | 0.00251 | NA | NA | 0.884 |
| GCST90274765 | CCL19 | Critical COVID-19 | WM | 37 | -0.014 (0.056) | 0.99 [0.88-1.10] | NA | NA | NA | NA | NA | 0.807 |
| GCST90274765 | CCL19 | Critical COVID-19 | MR-Egger | 37 | -0.043 (0.068) | 0.96 [0.84-1.10] | 35.65 | 0.437 | -0.00969 | 0.005 | 0.517 | 0.535 |
| GCST90274766 | CCL20 | Critical COVID-19 | IVW | 33 | -0.012 (0.052) | 0.99 [0.89-1.09] | 43.76 | 0.08 | 0.269 | NA | NA | 0.819 |
| GCST90274766 | CCL20 | Critical COVID-19 | WM | 33 | -0.015 (0.064) | 0.98 [0.87-1.12] | NA | NA | NA | NA | NA | 0.811 |
| GCST90274766 | CCL20 | Critical COVID-19 | MR-Egger | 33 | -0.264 (0.122) | 0.77 [0.60-0.98] | 37.65 | 0.191 | 0.15 | 0.025 | 0.032 | 0.039 |
| GCST90274767 | CCL23 | Critical COVID-19 | IVW | 35 | -0.009 (0.037) | 0.99 [0.92-1.07] | 50.46 | 0.034 | 0.326 | NA | NA | 0.812 |
| GCST90274767 | CCL23 | Critical COVID-19 | WM | 35 | -0.042 (0.039) | 0.96 [0.89-1.03] | NA | NA | NA | NA | NA | 0.282 |
| GCST90274767 | CCL23 | Critical COVID-19 | MR-Egger | 35 | -0.043 (0.053) | 0.96 [0.86-1.06] | 49.25 | 0.034 | 0.31 | 0.007 | 0.375 | 0.422 |
| GCST90274768 | CCL25 | Critical COVID-19 | IVW | 33 | -0.023 (0.040) | 0.98 [0.90-1.06] | 70.77 | 9.43E-05 | 0.548 | NA | NA | 0.562 |
| GCST90274768 | CCL25 | Critical COVID-19 | WM | 33 | 0.043 (0.037) | 1.04 [0.97-1.12] | NA | NA | NA | NA | NA | 0.246 |
| GCST90274768 | CCL25 | Critical COVID-19 | MR-Egger | 33 | -0.051 (0.063) | 0.95 [0.84-1.07] | 70.01 | 7.63E-05 | 0.543 | 0.005 | 0.565 | 0.421 |
| GCST90274769 | CCL28 | Critical COVID-19 | IVW | 38 | -0.080 (0.065) | 0.92 [0.81-1.05] | 83.56 | 1.87E-05 | 0.557 | NA | NA | 0.218 |
| GCST90274769 | CCL28 | Critical COVID-19 | WM | 38 | -0.044 (0.066) | 0.96 [0.84-1.09] | NA | NA | NA | NA | NA | 0.503 |
| GCST90274769 | CCL28 | Critical COVID-19 | MR-Egger | 38 | -0.143 (0.136) | 0.87 [0.66-1.13] | 82.92 | 1.45E-05 | 0.554 | 0.006 | 0.601 | 0.301 |
| GCST90274770 | CCL4 | Critical COVID-19 | IVW | 30 | -0.098 (0.034) | 0.91 [0.85-0.97] | 39.78 | 0.088 | 0.271 | NA | NA | 3.69E-03 |
| GCST90274770 | CCL4 | Critical COVID-19 | WM | 30 | -0.182 (0.039) | 0.83 [0.77-0.90] | NA | NA | NA | NA | NA | 3.19E-06 |
| GCST90274770 | CCL4 | Critical COVID-19 | MR-Egger | 30 | -0.171 (0.045) | 0.84 [0.77-0.92] | 33.52 | 0.217 | 0.135 | 0.017 | 0.03 | 6.95E-04 |
| GCST90274771 | CD244 | Critical COVID-19 | IVW | 32 | -0.061 (0.041) | 0.94 [0.87-1.02] | 37.8 | 0.187 | 0.18 | NA | NA | 0.137 |
| GCST90274771 | CD244 | Critical COVID-19 | WM | 32 | 0.020 (0.061) | 1.02 [0.91-1.15] | NA | NA | NA | NA | NA | 0.74 |
| GCST90274771 | CD244 | Critical COVID-19 | MR-Egger | 32 | 0.085 (0.079) | 1.09 [0.93-1.27] | 32.82 | 0.33 | 0.056 | -0.018 | 0.041 | 0.289 |
| GCST90274772 | CD40 | Critical COVID-19 | IVW | 24 | -0.056 (0.031) | 0.95 [0.89-1.00] | 11.56 | 0.977 | -0.99 | NA | NA | 0.071 |
| GCST90274772 | CD40 | Critical COVID-19 | WM | 24 | -0.077 (0.037) | 0.93 [0.86-1.00] | NA | NA | NA | NA | NA | 0.039 |
| GCST90274772 | CD40 | Critical COVID-19 | MR-Egger | 24 | -0.071 (0.045) | 0.93 [0.85-1.02] | 11.33 | 0.97 | -1.03 | 0.003 | 0.64 | 0.123 |
| GCST90274773 | CD5 | Critical COVID-19 | IVW | 32 | -0.023 (0.049) | 0.98 [0.89-1.08] | 42.41 | 0.083 | 0.269 | NA | NA | 0.642 |
| GCST90274773 | CD5 | Critical COVID-19 | WM | 32 | -0.064 (0.062) | 0.94 [0.83-1.06] | NA | NA | NA | NA | NA | 0.305 |
| GCST90274773 | CD5 | Critical COVID-19 | MR-Egger | 32 | -0.031 (0.121) | 0.97 [0.76-1.23] | 42.41 | 0.066 | 0.269 | 0.001 | 0.942 | 0.802 |
| GCST90274774 | CD6 | Critical COVID-19 | IVW | 27 | 0.010 (0.029) | 1.01 [0.95-1.07] | 30.02 | 0.267 | 0.134 | NA | NA | 0.734 |
| GCST90274774 | CD6 | Critical COVID-19 | WM | 27 | 0.039 (0.034) | 1.04 [0.97-1.11] | NA | NA | NA | NA | NA | 0.248 |
| GCST90274774 | CD6 | Critical COVID-19 | MR-Egger | 27 | 0.056 (0.040) | 1.06 [0.98-1.14] | 27.13 | 0.349 | 0.042 | -0.011 | 0.115 | 0.171 |
| GCST90274775 | CDCP1 | Critical COVID-19 | IVW | 33 | -0.125 (0.036) | 0.88 [0.82-0.95] | 29.58 | 0.589 | -0.082 | NA | NA | 4.27E-04 |
| GCST90274775 | CDCP1 | Critical COVID-19 | WM | 33 | -0.165 (0.055) | 0.85 [0.76-0.94] | NA | NA | NA | NA | NA | 2.43E-03 |
| GCST90274775 | CDCP1 | Critical COVID-19 | MR-Egger | 33 | -0.092 (0.066) | 0.91 [0.80-1.04] | 29.21 | 0.558 | -0.096 | -0.004 | 0.545 | 0.172 |
| GCST90274776 | CSF-1 | Critical COVID-19 | IVW | 29 | -0.018 (0.046) | 0.98 [0.90-1.08] | 21.16 | 0.819 | -0.323 | NA | NA | 0.702 |
| GCST90274776 | CSF-1 | Critical COVID-19 | WM | 29 | -0.028 (0.074) | 0.97 [0.84-1.12] | NA | NA | NA | NA | NA | 0.706 |
| GCST90274776 | CSF-1 | Critical COVID-19 | MR-Egger | 29 | -0.046 (0.106) | 0.95 [0.78-1.17] | 21.07 | 0.783 | -0.329 | 0.003 | 0.767 | 0.666 |
| GCST90274777 | CST5 | Critical COVID-19 | IVW | 47 | 0.054 (0.031) | 1.06 [0.99-1.12] | 61.51 | 0.063 | 0.252 | NA | NA | 0.075 |
| GCST90274777 | CST5 | Critical COVID-19 | WM | 47 | 0.058 (0.038) | 1.06 [0.98-1.14] | NA | NA | NA | NA | NA | 0.131 |
| GCST90274777 | CST5 | Critical COVID-19 | MR-Egger | 47 | 0.057 (0.049) | 1.06 [0.96-1.16] | 61.51 | 0.051 | 0.252 | 0 | 0.939 | 0.244 |
| GCST90274778 | CX3CL1 | Critical COVID-19 | IVW | 31 | 0.008 (0.081) | 1.01 [0.86-1.18] | 96.57 | 6.40E-09 | 0.689 | NA | NA | 0.925 |
| GCST90274778 | CX3CL1 | Critical COVID-19 | WM | 31 | -0.005 (0.070) | 1.00 [0.87-1.14] | NA | NA | NA | NA | NA | 0.947 |
| GCST90274778 | CX3CL1 | Critical COVID-19 | MR-Egger | 31 | -0.177 (0.214) | 0.84 [0.55-1.27] | 93.76 | 9.51E-09 | 0.68 | 0.018 | 0.358 | 0.414 |
| GCST90274779 | CXCL1 | Critical COVID-19 | IVW | 26 | 0.000 (0.050) | 1.00 [0.91-1.10] | 40.39 | 0.027 | 0.381 | NA | NA | 0.999 |
| GCST90274779 | CXCL1 | Critical COVID-19 | WM | 26 | 0.005 (0.052) | 1.01 [0.91-1.11] | NA | NA | NA | NA | NA | 0.916 |
| GCST90274779 | CXCL1 | Critical COVID-19 | MR-Egger | 26 | -0.040 (0.082) | 0.96 [0.82-1.13] | 39.73 | 0.023 | 0.371 | 0.006 | 0.534 | 0.626 |
| GCST90274780 | CXCL10 | Critical COVID-19 | IVW | 33 | 0.018 (0.057) | 1.02 [0.91-1.14] | 74.43 | 3.12E-05 | 0.57 | NA | NA | 0.75 |
| GCST90274780 | CXCL10 | Critical COVID-19 | WM | 33 | 0.039 (0.062) | 1.04 [0.92-1.17] | NA | NA | NA | NA | NA | 0.53 |
| GCST90274780 | CXCL10 | Critical COVID-19 | MR-Egger | 33 | 0.017 (0.101) | 1.02 [0.83-1.24] | 74.43 | 1.96E-05 | 0.57 | 0 | 0.99 | 0.869 |
| GCST90274781 | CXCL11 | Critical COVID-19 | IVW | 38 | -0.089 (0.039) | 0.92 [0.85-0.99] | 40.79 | 0.307 | 0.093 | NA | NA | 0.022 |
| GCST90274781 | CXCL11 | Critical COVID-19 | WM | 38 | -0.149 (0.061) | 0.86 [0.76-0.97] | NA | NA | NA | NA | NA | 0.015 |
| GCST90274781 | CXCL11 | Critical COVID-19 | MR-Egger | 38 | -0.135 (0.092) | 0.87 [0.73-1.05] | 40.45 | 0.28 | 0.085 | 0.005 | 0.584 | 0.152 |
| GCST90274782 | CXCL5 | Critical COVID-19 | IVW | 23 | 0.001 (0.036) | 1.00 [0.93-1.08] | 28.37 | 0.164 | 0.225 | NA | NA | 0.968 |
| GCST90274782 | CXCL5 | Critical COVID-19 | WM | 23 | -0.016 (0.040) | 0.98 [0.91-1.06] | NA | NA | NA | NA | NA | 0.691 |
| GCST90274782 | CXCL5 | Critical COVID-19 | MR-Egger | 23 | -0.066 (0.054) | 0.94 [0.84-1.04] | 25.19 | 0.239 | 0.127 | 0.012 | 0.118 | 0.237 |
| GCST90274783 | CXCL6 | Critical COVID-19 | IVW | 24 | -0.008 (0.034) | 0.99 [0.93-1.06] | 27.02 | 0.255 | 0.149 | NA | NA | 0.823 |
| GCST90274783 | CXCL6 | Critical COVID-19 | WM | 24 | -0.019 (0.037) | 0.98 [0.91-1.05] | NA | NA | NA | NA | NA | 0.604 |
| GCST90274783 | CXCL6 | Critical COVID-19 | MR-Egger | 24 | -0.034 (0.053) | 0.97 [0.87-1.07] | 26.5 | 0.231 | 0.132 | 0.005 | 0.518 | 0.528 |
| GCST90274784 | CXCL9 | Critical COVID-19 | IVW | 37 | -0.046 (0.061) | 0.96 [0.85-1.08] | 70.2 | 5.56E-04 | 0.487 | NA | NA | 0.45 |
| GCST90274784 | CXCL9 | Critical COVID-19 | WM | 37 | -0.020 (0.072) | 0.98 [0.85-1.13] | NA | NA | NA | NA | NA | 0.776 |
| GCST90274784 | CXCL9 | Critical COVID-19 | MR-Egger | 37 | -0.051 (0.139) | 0.95 [0.72-1.25] | 70.19 | 3.81E-04 | 0.487 | 0.001 | 0.965 | 0.714 |
| GCST90274785 | DNER | Critical COVID-19 | IVW | 27 | 0.026 (0.054) | 1.03 [0.92-1.14] | 41.44 | 0.028 | 0.373 | NA | NA | 0.63 |
| GCST90274785 | DNER | Critical COVID-19 | WM | 27 | -0.045 (0.063) | 0.96 [0.84-1.08] | NA | NA | NA | NA | NA | 0.472 |
| GCST90274785 | DNER | Critical COVID-19 | MR-Egger | 27 | 0.042 (0.115) | 1.04 [0.83-1.31] | 41.4 | 0.021 | 0.372 | -0.002 | 0.871 | 0.715 |
| GCST90274786 | EN-RAGE | Critical COVID-19 | IVW | 26 | 0.021 (0.049) | 1.02 [0.93-1.12] | 20.95 | 0.695 | -0.193 | NA | NA | 0.674 |
| GCST90274786 | EN-RAGE | Critical COVID-19 | WM | 26 | 0.004 (0.076) | 1.00 [0.87-1.16] | NA | NA | NA | NA | NA | 0.954 |
| GCST90274786 | EN-RAGE | Critical COVID-19 | MR-Egger | 26 | 0.033 (0.103) | 1.03 [0.84-1.27] | 20.93 | 0.643 | -0.194 | -0.001 | 0.896 | 0.756 |
| GCST90274787 | FGF-19 | Critical COVID-19 | IVW | 33 | 0.141 (0.054) | 1.15 [1.03-1.28] | 57.34 | 3.87E-03 | 0.442 | NA | NA | 9.64E-03 |
| GCST90274787 | FGF-19 | Critical COVID-19 | WM | 33 | 0.078 (0.069) | 1.08 [0.94-1.24] | NA | NA | NA | NA | NA | 0.259 |
| GCST90274787 | FGF-19 | Critical COVID-19 | MR-Egger | 33 | 0.377 (0.131) | 1.46 [1.13-1.88] | 50.95 | 0.013 | 0.372 | -0.029 | 0.058 | 7.06E-03 |
| GCST90274788 | FGF-21 | Critical COVID-19 | IVW | 26 | 0.003 (0.052) | 1.00 [0.91-1.11] | 38.81 | 0.038 | 0.356 | NA | NA | 0.952 |
| GCST90274788 | FGF-21 | Critical COVID-19 | WM | 26 | 0.019 (0.069) | 1.02 [0.89-1.17] | NA | NA | NA | NA | NA | 0.779 |
| GCST90274788 | FGF-21 | Critical COVID-19 | MR-Egger | 26 | -0.004 (0.117) | 1.00 [0.79-1.25] | 38.81 | 0.029 | 0.356 | 0.001 | 0.948 | 0.975 |
| GCST90274789 | FGF-23 | Critical COVID-19 | IVW | 26 | 0.030 (0.051) | 1.03 [0.93-1.14] | 22.08 | 0.631 | -0.132 | NA | NA | 0.565 |
| GCST90274789 | FGF-23 | Critical COVID-19 | WM | 26 | 0.078 (0.073) | 1.08 [0.94-1.25] | NA | NA | NA | NA | NA | 0.287 |
| GCST90274789 | FGF-23 | Critical COVID-19 | MR-Egger | 26 | -0.003 (0.133) | 1.00 [0.77-1.29] | 22.02 | 0.578 | -0.136 | 0.003 | 0.796 | 0.984 |
| GCST90274790 | FGF-5 | Critical COVID-19 | IVW | 33 | -0.031 (0.036) | 0.97 [0.90-1.04] | 36.48 | 0.268 | 0.123 | NA | NA | 0.377 |
| GCST90274790 | FGF-5 | Critical COVID-19 | WM | 33 | -0.048 (0.047) | 0.95 [0.87-1.05] | NA | NA | NA | NA | NA | 0.311 |
| GCST90274790 | FGF-5 | Critical COVID-19 | MR-Egger | 33 | -0.067 (0.056) | 0.94 [0.84-1.04] | 35.72 | 0.256 | 0.104 | 0.006 | 0.424 | 0.245 |
| GCST90274791 | FIt3L | Critical COVID-19 | IVW | 46 | 0.045 (0.031) | 1.05 [0.98-1.11] | 39.25 | 0.713 | -0.147 | NA | NA | 0.145 |
| GCST90274791 | FIt3L | Critical COVID-19 | WM | 46 | -0.007 (0.047) | 0.99 [0.91-1.09] | NA | NA | NA | NA | NA | 0.886 |
| GCST90274791 | FIt3L | Critical COVID-19 | MR-Egger | 46 | -0.041 (0.052) | 0.96 [0.87-1.06] | 35.07 | 0.83 | -0.283 | 0.011 | 0.047 | 0.438 |
| GCST90274792 | hGDNF | Critical COVID-19 | IVW | 25 | -0.037 (0.042) | 0.96 [0.89-1.05] | 25.61 | 0.373 | 0.063 | NA | NA | 0.377 |
| GCST90274792 | hGDNF | Critical COVID-19 | WM | 25 | -0.008 (0.061) | 0.99 [0.88-1.12] | NA | NA | NA | NA | NA | 0.89 |
| GCST90274792 | hGDNF | Critical COVID-19 | MR-Egger | 25 | 0.001 (0.074) | 1.00 [0.87-1.16] | 25.17 | 0.342 | 0.046 | -0.006 | 0.529 | 0.987 |
| GCST90274793 | HGF | Critical COVID-19 | IVW | 31 | -0.082 (0.046) | 0.92 [0.84-1.01] | 19.74 | 0.923 | -0.52 | NA | NA | 0.074 |
| GCST90274793 | HGF | Critical COVID-19 | WM | 31 | -0.060 (0.067) | 0.94 [0.83-1.07] | NA | NA | NA | NA | NA | 0.372 |
| GCST90274793 | HGF | Critical COVID-19 | MR-Egger | 31 | -0.074 (0.098) | 0.93 [0.77-1.12] | 19.73 | 0.901 | -0.52 | -0.001 | 0.927 | 0.452 |
| GCST90274794 | IFN-γ | Critical COVID-19 | IVW | 20 | -0.121 (0.054) | 0.89 [0.80-0.99] | 13.21 | 0.828 | -0.439 | NA | NA | 0.026 |
| GCST90274794 | IFNγ | Critical COVID-19 | WM | 20 | -0.141 (0.074) | 0.87 [0.75-1.00] | NA | NA | NA | NA | NA | 0.058 |
| GCST90274794 | IFN-γ | Critical COVID-19 | MR-Egger | 20 | -0.154 (0.099) | 0.86 [0.71-1.04] | 13.04 | 0.789 | -0.457 | 0.004 | 0.692 | 0.135 |
| GCST90274795 | IL-10 | Critical COVID-19 | IVW | 32 | 0.055 (0.043) | 1.06 [0.97-1.15] | 29.87 | 0.524 | -0.038 | NA | NA | 0.203 |
| GCST90274795 | IL-10 | Critical COVID-19 | WM | 32 | 0.041 (0.065) | 1.04 [0.92-1.18] | NA | NA | NA | NA | NA | 0.53 |
| GCST90274795 | IL-10 | Critical COVID-19 | MR-Egger | 32 | -0.017 (0.094) | 0.98 [0.82-1.18] | 29.12 | 0.511 | -0.064 | 0.008 | 0.395 | 0.856 |
| GCST90274796 | IL-10RA | Critical COVID-19 | IVW | 20 | 0.112 (0.065) | 1.12 [0.99-1.27] | 24.29 | 0.185 | 0.218 | NA | NA | 0.084 |
| GCST90274796 | IL-10RA | Critical COVID-19 | WM | 20 | 0.066 (0.087) | 1.07 [0.90-1.27] | NA | NA | NA | NA | NA | 0.449 |
| GCST90274796 | IL-10RA | Critical COVID-19 | MR-Egger | 20 | 0.023 (0.161) | 1.02 [0.75-1.40] | 23.81 | 0.161 | 0.202 | 0.009 | 0.555 | 0.887 |
| GCST90274797 | IL10RB | Critical COVID-19 | IVW | 29 | 0.147 (0.034) | 1.16 [1.08-1.24] | 38.45 | 0.09 | 0.272 | NA | NA | 1.67E-05 |
| GCST90274797 | IL10RB | Critical COVID-19 | WM | 29 | 0.161 (0.034) | 1.18 [1.10-1.26] | NA | NA | NA | NA | NA | 1.64E-06 |
| GCST90274797 | IL10RB | Critical COVID-19 | MR-Egger | 29 | 0.152 (0.049) | 1.16 [1.06-1.28] | 38.43 | 0.071 | 0.271 | -0.001 | 0.896 | 4.39E-03 |
| GCST90274798 | IL-12B | Critical COVID-19 | IVW | 37 | -0.013 (0.023) | 0.99 [0.94-1.03] | 38.34 | 0.364 | 0.061 | NA | NA | 0.574 |
| GCST90274798 | IL-12B | Critical COVID-19 | WM | 37 | -0.038 (0.033) | 0.96 [0.90-1.03] | NA | NA | NA | NA | NA | 0.247 |
| GCST90274798 | IL-12B | Critical COVID-19 | MR-Egger | 37 | -0.011 (0.037) | 0.99 [0.92-1.06] | 38.33 | 0.321 | 0.061 | -0.001 | 0.929 | 0.776 |
| GCST90274799 | IL-13 | Critical COVID-19 | IVW | 28 | 0.007 (0.053) | 1.01 [0.91-1.12] | 32.11 | 0.228 | 0.159 | NA | NA | 0.891 |
| GCST90274799 | IL-13 | Critical COVID-19 | WM | 28 | 0.034 (0.069) | 1.03 [0.90-1.18] | NA | NA | NA | NA | NA | 0.627 |
| GCST90274799 | IL-13 | Critical COVID-19 | MR-Egger | 28 | 0.079 (0.136) | 1.08 [0.83-1.41] | 31.71 | 0.203 | 0.148 | -0.007 | 0.571 | 0.566 |
| GCST90274800 | IL-15RA | Critical COVID-19 | IVW | 23 | 0.023 (0.029) | 1.02 [0.97-1.08] | 22.44 | 0.434 | 0.02 | NA | NA | 0.433 |
| GCST90274800 | IL-15RA | Critical COVID-19 | WM | 23 | 0.015 (0.035) | 1.02 [0.95-1.09] | NA | NA | NA | NA | NA | 0.665 |
| GCST90274800 | IL-15RA | Critical COVID-19 | MR-Egger | 23 | 0.028 (0.048) | 1.03 [0.94-1.13] | 22.41 | 0.376 | 0.018 | -0.001 | 0.88 | 0.562 |
| GCST90274801 | IL-17A | Critical COVID-19 | IVW | 22 | -0.040 (0.062) | 0.96 [0.85-1.08] | 30.15 | 0.089 | 0.303 | NA | NA | 0.515 |
| GCST90274801 | IL-17A | Critical COVID-19 | WM | 22 | 0.010 (0.080) | 1.01 [0.86-1.18] | NA | NA | NA | NA | NA | 0.901 |
| GCST90274801 | IL-17A | Critical COVID-19 | MR-Egger | 22 | -0.156 (0.135) | 0.86 [0.66-1.11] | 28.81 | 0.092 | 0.271 | 0.013 | 0.346 | 0.261 |
| GCST90274802 | IL-17C | Critical COVID-19 | IVW | 37 | -0.035 (0.046) | 0.97 [0.88-1.06] | 46.94 | 0.105 | 0.233 | NA | NA | 0.445 |
| GCST90274802 | IL-17C | Critical COVID-19 | WM | 37 | -0.095 (0.060) | 0.91 [0.81-1.02] | NA | NA | NA | NA | NA | 0.113 |
| GCST90274802 | IL-17C | Critical COVID-19 | MR-Egger | 37 | -0.072 (0.110) | 0.93 [0.75-1.15] | 46.76 | 0.088 | 0.23 | 0.004 | 0.713 | 0.517 |
| GCST90274803 | IL-18 | Critical COVID-19 | IVW | 32 | -0.069 (0.041) | 0.93 [0.86-1.01] | 36.51 | 0.228 | 0.151 | NA | NA | 0.088 |
| GCST90274803 | IL-18 | Critical COVID-19 | WM | 32 | -0.036 (0.053) | 0.96 [0.87-1.07] | NA | NA | NA | NA | NA | 0.504 |
| GCST90274803 | IL-18 | Critical COVID-19 | MR-Egger | 32 | -0.139 (0.084) | 0.87 [0.74-1.03] | 35.44 | 0.227 | 0.125 | 0.009 | 0.351 | 0.109 |
| GCST90274804 | IL-18R1 | Critical COVID-19 | IVW | 38 | -0.017 (0.023) | 0.98 [0.94-1.03] | 30.22 | 0.777 | -0.224 | NA | NA | 0.464 |
| GCST90274804 | IL-18R1 | Critical COVID-19 | WM | 38 | -0.021 (0.030) | 0.98 [0.92-1.04] | NA | NA | NA | NA | NA | 0.488 |
| GCST90274804 | IL-18R1 | Critical COVID-19 | MR-Egger | 38 | -0.030 (0.034) | 0.97 [0.91-1.04] | 29.95 | 0.751 | -0.235 | 0.003 | 0.608 | 0.385 |
| GCST90274805 | IL-1α | Critical COVID-19 | IVW | 22 | 0.015 (0.055) | 1.02 [0.91-1.13] | 26.32 | 0.195 | 0.202 | NA | NA | 0.782 |
| GCST90274805 | IL-1α | Critical COVID-19 | WM | 22 | 0.015 (0.074) | 1.01 [0.88-1.17] | NA | NA | NA | NA | NA | 0.842 |
| GCST90274805 | IL-1α | Critical COVID-19 | MR-Egger | 22 | 0.033 (0.117) | 1.03 [0.82-1.30] | 26.28 | 0.157 | 0.201 | -0.002 | 0.868 | 0.784 |
| GCST90274806 | IL-2 | Critical COVID-19 | IVW | 21 | 0.081 (0.059) | 1.08 [0.97-1.22] | 22.76 | 0.301 | 0.121 | NA | NA | 0.171 |
| GCST90274806 | IL-2 | Critical COVID-19 | WM | 21 | 0.046 (0.081) | 1.05 [0.89-1.23] | NA | NA | NA | NA | NA | 0.576 |
| GCST90274806 | IL-2 | Critical COVID-19 | MR-Egger | 21 | 0.225 (0.147) | 1.25 [0.94-1.67] | 21.47 | 0.311 | 0.068 | -0.015 | 0.3 | 0.143 |
| GCST90274807 | IL-20 | Critical COVID-19 | IVW | 23 | 0.067 (0.053) | 1.07 [0.96-1.19] | 19.77 | 0.597 | -0.113 | NA | NA | 0.204 |
| GCST90274807 | IL-20 | Critical COVID-19 | WM | 23 | 0.094 (0.075) | 1.10 [0.95-1.27] | NA | NA | NA | NA | NA | 0.21 |
| GCST90274807 | IL-20 | Critical COVID-19 | MR-Egger | 23 | -0.077 (0.115) | 0.93 [0.74-1.16] | 17.79 | 0.662 | -0.236 | 0.021 | 0.174 | 0.513 |
| GCST90274808 | IL-20RA | Critical COVID-19 | IVW | 21 | -0.076 (0.052) | 0.93 [0.84-1.03] | 12.84 | 0.884 | -0.558 | NA | NA | 0.142 |
| GCST90274808 | IL-20RA | Critical COVID-19 | WM | 21 | -0.076 (0.070) | 0.93 [0.81-1.06] | NA | NA | NA | NA | NA | 0.277 |
| GCST90274808 | IL-20RA | Critical COVID-19 | MR-Egger | 21 | -0.103 (0.113) | 0.90 [0.72-1.13] | 12.77 | 0.85 | -0.566 | 0.003 | 0.796 | 0.376 |
| GCST90274809 | IL-22RA1 | Critical COVID-19 | IVW | 21 | -0.108 (0.059) | 0.90 [0.80-1.01] | 23.46 | 0.267 | 0.147 | NA | NA | 0.065 |
| GCST90274809 | IL-22RA1 | Critical COVID-19 | WM | 21 | -0.138 (0.082) | 0.87 [0.74-1.02] | NA | NA | NA | NA | NA | 0.094 |
| GCST90274809 | IL-22RA1 | Critical COVID-19 | MR-Egger | 21 | -0.121 (0.115) | 0.89 [0.71-1.11] | 23.43 | 0.219 | 0.147 | 0.002 | 0.893 | 0.305 |
| GCST90274810 | IL-24 | Critical COVID-19 | IVW | 19 | -0.075 (0.070) | 0.93 [0.81-1.07] | 29.07 | 0.047 | 0.381 | NA | NA | 0.289 |
| GCST90274810 | IL-24 | Critical COVID-19 | WM | 19 | -0.157 (0.080) | 0.85 [0.73-1.00] | NA | NA | NA | NA | NA | 0.049 |
| GCST90274810 | IL-24 | Critical COVID-19 | MR-Egger | 19 | 0.203 (0.129) | 1.22 [0.95-1.58] | 21.48 | 0.206 | 0.162 | -0.033 | 0.025 | 0.135 |
| GCST90274811 | IL-2RB | Critical COVID-19 | IVW | 22 | -0.063 (0.053) | 0.94 [0.85-1.04] | 15.66 | 0.789 | -0.341 | NA | NA | 0.235 |
| GCST90274811 | IL-2RB | Critical COVID-19 | WM | 22 | -0.015 (0.070) | 0.99 [0.86-1.13] | NA | NA | NA | NA | NA | 0.832 |
| GCST90274811 | IL-2RB | Critical COVID-19 | MR-Egger | 22 | -0.210 (0.110) | 0.81 [0.65-1.01] | 13.34 | 0.862 | -0.574 | 0.017 | 0.144 | 0.071 |
| GCST90274812 | IL-33 | Critical COVID-19 | IVW | 22 | 0.037 (0.054) | 1.04 [0.93-1.15] | 12.31 | 0.931 | -0.706 | NA | NA | 0.489 |
| GCST90274812 | IL-33 | Critical COVID-19 | WM | 22 | 0.063 (0.071) | 1.06 [0.93-1.22] | NA | NA | NA | NA | NA | 0.377 |
| GCST90274812 | IL-33 | Critical COVID-19 | MR-Egger | 22 | -0.019 (0.141) | 0.98 [0.74-1.29] | 12.12 | 0.912 | -0.733 | 0.006 | 0.668 | 0.892 |
| GCST90274813 | IL-4 | Critical COVID-19 | IVW | 21 | 0.054 (0.065) | 1.06 [0.93-1.20] | 29.72 | 0.075 | 0.327 | NA | NA | 0.403 |
| GCST90274813 | IL-4 | Critical COVID-19 | WM | 21 | 0.058 (0.083) | 1.06 [0.90-1.25] | NA | NA | NA | NA | NA | 0.483 |
| GCST90274813 | IL-4 | Critical COVID-19 | MR-Egger | 21 | -0.313 (0.146) | 0.73 [0.55-0.97] | 21.32 | 0.319 | 0.062 | 0.036 | 0.013 | 0.045 |
| GCST90274814 | IL-5 | Critical COVID-19 | IVW | 22 | 0.002 (0.050) | 1.00 [0.91-1.11] | 17.13 | 0.703 | -0.226 | NA | NA | 0.961 |
| GCST90274814 | IL-5 | Critical COVID-19 | WM | 22 | 0.003 (0.066) | 1.00 [0.88-1.14] | NA | NA | NA | NA | NA | 0.965 |
| GCST90274814 | IL-5 | Critical COVID-19 | MR-Egger | 22 | -0.041 (0.116) | 0.96 [0.76-1.21] | 16.96 | 0.656 | -0.238 | 0.005 | 0.684 | 0.729 |
| GCST90274815 | IL-6 | Critical COVID-19 | IVW | 14 | -0.060 (0.067) | 0.94 [0.83-1.08] | 18.1 | 0.154 | 0.282 | NA | NA | 0.376 |
| GCST90274815 | IL-6 | Critical COVID-19 | WM | 14 | -0.186 (0.082) | 0.83 [0.71-0.97] | NA | NA | NA | NA | NA | 0.023 |
| GCST90274815 | IL-6 | Critical COVID-19 | MR-Egger | 14 | -0.056 (0.140) | 0.95 [0.72-1.24] | 18.09 | 0.113 | 0.282 | 0 | 0.978 | 0.695 |
| GCST90274816 | IL-7 | Critical COVID-19 | IVW | 23 | 0.150 (0.066) | 1.16 [1.02-1.32] | 34.3 | 0.046 | 0.359 | NA | NA | 0.023 |
| GCST90274816 | IL-7 | Critical COVID-19 | WM | 23 | 0.086 (0.079) | 1.09 [0.93-1.27] | NA | NA | NA | NA | NA | 0.276 |
| GCST90274816 | IL-7 | Critical COVID-19 | MR-Egger | 23 | -0.015 (0.164) | 0.99 [0.71-1.36] | 32.42 | 0.053 | 0.321 | 0.017 | 0.283 | 0.927 |
| GCST90274817 | IL-8 | Critical COVID-19 | IVW | 28 | -0.028 (0.055) | 0.97 [0.87-1.08] | 32.65 | 0.209 | 0.173 | NA | NA | 0.609 |
| GCST90274817 | IL-8 | Critical COVID-19 | WM | 28 | 0.022 (0.074) | 1.02 [0.88-1.18] | NA | NA | NA | NA | NA | 0.765 |
| GCST90274817 | IL-8 | Critical COVID-19 | MR-Egger | 28 | -0.027 (0.116) | 0.97 [0.78-1.22] | 32.65 | 0.172 | 0.173 | 0 | 0.986 | 0.821 |
| GCST90274818 | LAP TGF-β-1 | Critical COVID-19 | IVW | 29 | -0.012 (0.045) | 0.99 [0.90-1.08] | 23.53 | 0.706 | -0.19 | NA | NA | 0.79 |
| GCST90274818 | LAP TGF-β-1 | Critical COVID-19 | WM | 29 | -0.023 (0.072) | 0.98 [0.85-1.12] | NA | NA | NA | NA | NA | 0.745 |
| GCST90274818 | LAP TGF-β-1 | Critical COVID-19 | MR-Egger | 29 | 0.011 (0.079) | 1.01 [0.87-1.18] | 23.4 | 0.663 | -0.196 | -0.003 | 0.726 | 0.891 |
| GCST90274819 | LIF | Critical COVID-19 | IVW | 27 | 0.098 (0.047) | 1.10 [1.01-1.21] | 26.35 | 0.444 | 0.013 | NA | NA | 0.038 |
| GCST90274819 | LIF | Critical COVID-19 | WM | 27 | 0.061 (0.070) | 1.06 [0.93-1.22] | NA | NA | NA | NA | NA | 0.389 |
| GCST90274819 | LIF | Critical COVID-19 | MR-Egger | 27 | 0.081 (0.101) | 1.08 [0.89-1.32] | 26.32 | 0.391 | 0.012 | 0.002 | 0.853 | 0.432 |
| GCST90274820 | LIF-R | Critical COVID-19 | IVW | 29 | -0.164 (0.053) | 0.85 [0.76-0.94] | 55.16 | 1.63E-03 | 0.492 | NA | NA | 1.99E-03 |
| GCST90274820 | LIF-R | Critical COVID-19 | WM | 29 | -0.180 (0.074) | 0.84 [0.72-0.97] | NA | NA | NA | NA | NA | 0.015 |
| GCST90274820 | LIF-R | Critical COVID-19 | MR-Egger | 29 | -0.355 (0.084) | 0.70 [0.60-0.83] | 43 | 0.026 | 0.349 | 0.025 | 0.01 | 2.40E-04 |
| GCST90274821 | CCL2 | Critical COVID-19 | IVW | 30 | 0.123 (0.058) | 1.13 [1.01-1.27] | 60.8 | 4.90E-04 | 0.523 | NA | NA | 0.033 |
| GCST90274821 | CCL2 | Critical COVID-19 | WM | 30 | 0.075 (0.062) | 1.08 [0.95-1.22] | NA | NA | NA | NA | NA | 0.23 |
| GCST90274821 | CCL2 | Critical COVID-19 | MR-Egger | 30 | 0.225 (0.111) | 1.25 [1.01-1.56] | 58.43 | 6.44E-04 | 0.504 | -0.014 | 0.295 | 0.053 |
| GCST90274822 | CCL8 | Critical COVID-19 | IVW | 46 | -0.006 (0.021) | 0.99 [0.95-1.04] | 49.29 | 0.306 | 0.087 | NA | NA | 0.766 |
| GCST90274822 | CCL8 | Critical COVID-19 | WM | 46 | 0.012 (0.031) | 1.01 [0.95-1.08] | NA | NA | NA | NA | NA | 0.696 |
| GCST90274822 | CCL8 | Critical COVID-19 | MR-Egger | 46 | 0.018 (0.026) | 1.02 [0.97-1.07] | 46.71 | 0.362 | 0.037 | -0.008 | 0.126 | 0.487 |
| GCST90274823 | CCL7 | Critical COVID-19 | IVW | 27 | -0.059 (0.041) | 0.94 [0.87-1.02] | 14.99 | 0.957 | -0.734 | NA | NA | 0.151 |
| GCST90274823 | CCL7 | Critical COVID-19 | WM | 27 | -0.096 (0.061) | 0.91 [0.81-1.02] | NA | NA | NA | NA | NA | 0.114 |
| GCST90274823 | CCL7 | Critical COVID-19 | MR-Egger | 27 | -0.072 (0.093) | 0.93 [0.77-1.12] | 14.97 | 0.942 | -0.737 | 0.002 | 0.884 | 0.449 |
| GCST90274824 | CCL13 | Critical COVID-19 | IVW | 29 | -0.001 (0.033) | 1.00 [0.94-1.07] | 21.99 | 0.782 | -0.273 | NA | NA | 0.986 |
| GCST90274824 | CCL13 | Critical COVID-19 | WM | 29 | 0.041 (0.051) | 1.04 [0.94-1.15] | NA | NA | NA | NA | NA | 0.419 |
| GCST90274824 | CCL13 | Critical COVID-19 | MR-Egger | 29 | -0.001 (0.066) | 1.00 [0.88-1.14] | 21.99 | 0.738 | -0.273 | 0 | 0.999 | 0.992 |
| GCST90274825 | MIP-1α | Critical COVID-19 | IVW | 21 | -0.029 (0.039) | 0.97 [0.90-1.05] | 28.07 | 0.108 | 0.287 | NA | NA | 0.455 |
| GCST90274825 | MIP-1α | Critical COVID-19 | WM | 21 | -0.007 (0.040) | 0.99 [0.92-1.07] | NA | NA | NA | NA | NA | 0.854 |
| GCST90274825 | MIP-1α | Critical COVID-19 | MR-Egger | 21 | -0.007 (0.061) | 0.99 [0.88-1.12] | 27.74 | 0.089 | 0.279 | -0.004 | 0.641 | 0.91 |
| GCST90274826 | MMP-1 | Critical COVID-19 | IVW | 25 | -0.023 (0.045) | 0.98 [0.89-1.07] | 26.7 | 0.319 | 0.101 | NA | NA | 0.608 |
| GCST90274826 | MMP-1 | Critical COVID-19 | WM | 25 | 0.003 (0.062) | 1.00 [0.89-1.13] | NA | NA | NA | NA | NA | 0.957 |
| GCST90274826 | MMP-1 | Critical COVID-19 | MR-Egger | 25 | -0.002 (0.086) | 1.00 [0.84-1.18] | 26.61 | 0.273 | 0.098 | -0.003 | 0.778 | 0.98 |
| GCST90274827 | MMP-10 | Critical COVID-19 | IVW | 24 | -0.026 (0.030) | 0.97 [0.92-1.03] | 21.56 | 0.547 | -0.067 | NA | NA | 0.388 |
| GCST90274827 | MMP-10 | Critical COVID-19 | WM | 24 | -0.010 (0.040) | 0.99 [0.92-1.07] | NA | NA | NA | NA | NA | 0.811 |
| GCST90274827 | MMP-10 | Critical COVID-19 | MR-Egger | 24 | -0.073 (0.040) | 0.93 [0.86-1.01] | 18.32 | 0.687 | -0.255 | 0.013 | 0.086 | 0.08 |
| GCST90274828 | NRTN | Critical COVID-19 | IVW | 23 | 0.039 (0.059) | 1.04 [0.93-1.17] | 27.22 | 0.203 | 0.192 | NA | NA | 0.508 |
| GCST90274828 | NRTN | Critical COVID-19 | WM | 23 | 0.063 (0.077) | 1.07 [0.92-1.24] | NA | NA | NA | NA | NA | 0.413 |
| GCST90274828 | NRTN | Critical COVID-19 | MR-Egger | 23 | 0.078 (0.127) | 1.08 [0.84-1.39] | 27.07 | 0.169 | 0.187 | -0.004 | 0.733 | 0.547 |
| GCST90274829 | NT-3 | Critical COVID-19 | IVW | 31 | 0.117 (0.049) | 1.12 [1.02-1.24] | 25.04 | 0.723 | -0.198 | NA | NA | 0.016 |
| GCST90274829 | NT-3 | Critical COVID-19 | WM | 31 | 0.125 (0.070) | 1.13 [0.99-1.30] | NA | NA | NA | NA | NA | 0.074 |
| GCST90274829 | NT-3 | Critical COVID-19 | MR-Egger | 31 | 0.188 (0.105) | 1.21 [0.98-1.48] | 24.46 | 0.706 | -0.227 | -0.008 | 0.452 | 0.084 |
| GCST90274830 | OPG | Critical COVID-19 | IVW | 31 | -0.010 (0.051) | 0.99 [0.90-1.09] | 50.17 | 0.012 | 0.402 | NA | NA | 0.847 |
| GCST90274830 | OPG | Critical COVID-19 | WM | 31 | -0.084 (0.062) | 0.92 [0.81-1.04] | NA | NA | NA | NA | NA | 0.171 |
| GCST90274830 | OPG | Critical COVID-19 | MR-Egger | 31 | -0.027 (0.109) | 0.97 [0.79-1.20] | 50.11 | 8.78E-03 | 0.401 | 0.002 | 0.86 | 0.807 |
| GCST90274831 | OSM | Critical COVID-19 | IVW | 24 | -0.065 (0.069) | 0.94 [0.82-1.07] | 42.34 | 8.29E-03 | 0.457 | NA | NA | 0.345 |
| GCST90274831 | OSM | Critical COVID-19 | WM | 24 | -0.084 (0.083) | 0.92 [0.78-1.08] | NA | NA | NA | NA | NA | 0.308 |
| GCST90274831 | OSM | Critical COVID-19 | MR-Egger | 24 | -0.037 (0.188) | 0.96 [0.67-1.39] | 42.29 | 5.77E-03 | 0.456 | -0.003 | 0.874 | 0.844 |
| GCST90274832 | PD-L1 | Critical COVID-19 | IVW | 30 | 0.010 (0.054) | 1.01 [0.91-1.12] | 35.27 | 0.196 | 0.178 | NA | NA | 0.861 |
| GCST90274832 | PD-L1 | Critical COVID-19 | WM | 30 | 0.062 (0.073) | 1.06 [0.92-1.23] | NA | NA | NA | NA | NA | 0.399 |
| GCST90274832 | PD-L1 | Critical COVID-19 | MR-Egger | 30 | 0.009 (0.122) | 1.01 [0.79-1.28] | 35.27 | 0.162 | 0.178 | 0 | 0.997 | 0.941 |
| GCST90274833 | SCF | Critical COVID-19 | IVW | 45 | -0.005 (0.033) | 0.99 [0.93-1.06] | 51.93 | 0.192 | 0.153 | NA | NA | 0.874 |
| GCST90274833 | SCF | Critical COVID-19 | WM | 45 | 0.012 (0.054) | 1.01 [0.91-1.12] | NA | NA | NA | NA | NA | 0.83 |
| GCST90274833 | SCF | Critical COVID-19 | MR-Egger | 45 | 0.006 (0.067) | 1.01 [0.88-1.15] | 51.89 | 0.166 | 0.152 | -0.001 | 0.848 | 0.929 |
| GCST90274834 | SIRT2 | Critical COVID-19 | IVW | 19 | -0.022 (0.060) | 0.98 [0.87-1.10] | 17.59 | 0.483 | -0.023 | NA | NA | 0.713 |
| GCST90274834 | SIRT2 | Critical COVID-19 | WM | 19 | 0.002 (0.085) | 1.00 [0.85-1.18] | NA | NA | NA | NA | NA | 0.977 |
| GCST90274834 | SIRT2 | Critical COVID-19 | MR-Egger | 19 | -0.115 (0.133) | 0.89 [0.69-1.16] | 16.98 | 0.456 | -0.06 | 0.01 | 0.444 | 0.399 |
| GCST90274835 | SLAMF1 | Critical COVID-19 | IVW | 39 | -0.029 (0.045) | 0.97 [0.89-1.06] | 54.9 | 0.037 | 0.308 | NA | NA | 0.519 |
| GCST90274835 | SLAMF1 | Critical COVID-19 | WM | 39 | 0.060 (0.057) | 1.06 [0.95-1.19] | NA | NA | NA | NA | NA | 0.291 |
| GCST90274835 | SLAMF1 | Critical COVID-19 | MR-Egger | 39 | -0.079 (0.102) | 0.92 [0.76-1.13] | 54.46 | 0.032 | 0.302 | 0.005 | 0.59 | 0.445 |
| GCST90274836 | ST1A1 | Critical COVID-19 | IVW | 32 | -0.028 (0.043) | 0.97 [0.89-1.06] | 34.86 | 0.289 | 0.111 | NA | NA | 0.518 |
| GCST90274836 | ST1A1 | Critical COVID-19 | WM | 32 | -0.017 (0.062) | 0.98 [0.87-1.11] | NA | NA | NA | NA | NA | 0.788 |
| GCST90274836 | ST1A1 | Critical COVID-19 | MR-Egger | 32 | 0.054 (0.108) | 1.06 [0.85-1.30] | 34.07 | 0.278 | 0.09 | -0.009 | 0.409 | 0.617 |
| GCST90274837 | STAMPB | Critical COVID-19 | IVW | 21 | 0.018 (0.067) | 1.02 [0.89-1.16] | 25.69 | 0.176 | 0.221 | NA | NA | 0.784 |
| GCST90274837 | STAMPB | Critical COVID-19 | WM | 21 | 0.004 (0.085) | 1.00 [0.85-1.19] | NA | NA | NA | NA | NA | 0.961 |
| GCST90274837 | STAMPB | Critical COVID-19 | MR-Egger | 21 | -0.011 (0.163) | 0.99 [0.72-1.36] | 25.63 | 0.141 | 0.22 | 0.003 | 0.842 | 0.945 |
| GCST90274838 | TGF-α | Critical COVID-19 | IVW | 27 | 0.005 (0.070) | 1.00 [0.88-1.15] | 51.42 | 2.12E-03 | 0.494 | NA | NA | 0.945 |
| GCST90274838 | TGF-α | Critical COVID-19 | WM | 27 | 0.019 (0.076) | 1.02 [0.88-1.18] | NA | NA | NA | NA | NA | 0.799 |
| GCST90274838 | TGF-α | Critical COVID-19 | MR-Egger | 27 | 0.226 (0.155) | 1.25 [0.92-1.70] | 46.74 | 5.26E-03 | 0.444 | -0.024 | 0.126 | 0.158 |
| GCST90274839 | TNF | Critical COVID-19 | IVW | 29 | -0.020 (0.045) | 0.98 [0.90-1.07] | 23.33 | 0.716 | -0.2 | NA | NA | 0.649 |
| GCST90274839 | TNF | Critical COVID-19 | WM | 29 | -0.056 (0.061) | 0.95 [0.84-1.07] | NA | NA | NA | NA | NA | 0.361 |
| GCST90274839 | TNF | Critical COVID-19 | MR-Egger | 29 | -0.066 (0.093) | 0.94 [0.78-1.12] | 23.02 | 0.684 | -0.216 | 0.005 | 0.582 | 0.485 |
| GCST90274840 | TNFB | Critical COVID-19 | IVW | 42 | 0.014 (0.027) | 1.01 [0.96-1.07] | 47.97 | 0.211 | 0.145 | NA | NA | 0.594 |
| GCST90274840 | TNFB | Critical COVID-19 | WM | 42 | 0.081 (0.036) | 1.08 [1.01-1.16] | NA | NA | NA | NA | NA | 0.024 |
| GCST90274840 | TNFB | Critical COVID-19 | MR-Egger | 42 | 0.057 (0.042) | 1.06 [0.98-1.15] | 45.97 | 0.239 | 0.108 | -0.01 | 0.194 | 0.181 |
| GCST90274841 | TNFRSF9 | Critical COVID-19 | IVW | 34 | -0.041 (0.039) | 0.96 [0.89-1.04] | 26.14 | 0.796 | -0.262 | NA | NA | 0.294 |
| GCST90274841 | TNFRSF9 | Critical COVID-19 | WM | 34 | -0.009 (0.060) | 0.99 [0.88-1.11] | NA | NA | NA | NA | NA | 0.874 |
| GCST90274841 | TNFRSF9 | Critical COVID-19 | MR-Egger | 34 | 0.043 (0.086) | 1.04 [0.88-1.24] | 24.94 | 0.808 | -0.323 | -0.01 | 0.282 | 0.621 |
| GCST90274842 | TNFSF14 | Critical COVID-19 | IVW | 39 | -0.009 (0.034) | 0.99 [0.93-1.06] | 39.69 | 0.395 | 0.043 | NA | NA | 0.777 |
| GCST90274842 | TNFSF14 | Critical COVID-19 | WM | 39 | 0.052 (0.054) | 1.05 [0.95-1.17] | NA | NA | NA | NA | NA | 0.337 |
| GCST90274842 | TNFSF14 | Critical COVID-19 | MR-Egger | 39 | 0.052 (0.052) | 1.05 [0.95-1.17] | 37.43 | 0.449 | -0.015 | -0.01 | 0.144 | 0.333 |
| GCST90274843 | TRAIL | Critical COVID-19 | IVW | 37 | -0.021 (0.029) | 0.98 [0.93-1.04] | 24.95 | 0.917 | -0.443 | NA | NA | 0.465 |
| GCST90274843 | TRAIL | Critical COVID-19 | WM | 37 | -0.040 (0.043) | 0.96 [0.88-1.05] | NA | NA | NA | NA | NA | 0.356 |
| GCST90274843 | TRAIL | Critical COVID-19 | MR-Egger | 37 | -0.072 (0.046) | 0.93 [0.85-1.02] | 23.02 | 0.94 | -0.564 | 0.01 | 0.173 | 0.132 |
| GCST90274844 | TRANCE | Critical COVID-19 | IVW | 47 | 0.046 (0.032) | 1.05 [0.98-1.11] | 41.18 | 0.674 | -0.117 | NA | NA | 0.15 |
| GCST90274844 | TRANCE | Critical COVID-19 | WM | 47 | 0.027 (0.050) | 1.03 [0.93-1.13] | NA | NA | NA | NA | NA | 0.583 |
| GCST90274844 | TRANCE | Critical COVID-19 | MR-Egger | 47 | 0.070 (0.063) | 1.07 [0.95-1.21] | 40.97 | 0.643 | -0.123 | -0.003 | 0.652 | 0.269 |
| GCST90274845 | TSLP | Critical COVID-19 | IVW | 24 | 0.008 (0.059) | 1.01 [0.90-1.13] | 29.43 | 0.166 | 0.219 | NA | NA | 0.898 |
| GCST90274845 | TSLP | Critical COVID-19 | WM | 24 | 0.005 (0.074) | 1.01 [0.87-1.16] | NA | NA | NA | NA | NA | 0.943 |
| GCST90274845 | TSLP | Critical COVID-19 | MR-Egger | 24 | -0.105 (0.127) | 0.90 [0.70-1.15] | 28.16 | 0.17 | 0.183 | 0.015 | 0.33 | 0.419 |
| GCST90274846 | TWEAK | Critical COVID-19 | IVW | 43 | -0.027 (0.052) | 0.97 [0.88-1.08] | 97.61 | 2.55E-06 | 0.57 | NA | NA | 0.599 |
| GCST90274846 | TWEAK | Critical COVID-19 | WM | 43 | 0.000 (0.061) | 1.00 [0.89-1.13] | NA | NA | NA | NA | NA | 0.998 |
| GCST90274846 | TWEAK | Critical COVID-19 | MR-Egger | 43 | 0.164 (0.103) | 1.18 [0.96-1.44] | 87.96 | 2.84E-05 | 0.522 | -0.021 | 0.04 | 0.119 |
| GCST90274847 | uPA | Critical COVID-19 | IVW | 44 | -0.033 (0.038) | 0.97 [0.90-1.04] | 53.23 | 0.136 | 0.192 | NA | NA | 0.38 |
| GCST90274847 | uPA | Critical COVID-19 | WM | 44 | -0.057 (0.052) | 0.94 [0.85-1.05] | NA | NA | NA | NA | NA | 0.279 |
| GCST90274847 | uPA | Critical COVID-19 | MR-Egger | 44 | -0.135 (0.084) | 0.87 [0.74-1.03] | 50.97 | 0.161 | 0.156 | 0.011 | 0.18 | 0.114 |
| GCST90274848 | VEGF_A | Critical COVID-19 | IVW | 33 | -0.016 (0.031) | 0.98 [0.93-1.05] | 33.55 | 0.392 | 0.046 | NA | NA | 0.598 |
| GCST90274848 | VEGF_A | Critical COVID-19 | WM | 33 | 0.021 (0.041) | 1.02 [0.94-1.11] | NA | NA | NA | NA | NA | 0.61 |
| GCST90274848 | VEGF_A | Critical COVID-19 | MR-Egger | 33 | 0.064 (0.051) | 1.07 [0.97-1.18] | 29.64 | 0.536 | -0.08 | -0.014 | 0.057 | 0.215 |

OR: odds ratio; CI: confidence interval; b: effect size; se: standard error; N_IV: number of instrumental variables.
